# Evaluation of free radical scavenging and anxiolytic activities of *Bunium persicum* (Boiss.) B. Fedtsch. bioactive metabolites through multi-method experimental and computational approaches

**DOI:** 10.3389/fphar.2026.1805095

**Published:** 2026-06-05

**Authors:** Riehana Gani, Umar Yousuf, Noimul Hasan Siddiquee, Mushtaq A. Mir, Aadil Yaseen, Nasreena Bashir, Venkatramanan Varadharajan, Md. Ifteker Hossain, Pervaize Ahmad Dar, Mohd Younis Dar, Zulfiqar Ali Bhat, Basharat Ahmad Bhat

**Affiliations:** 1 Regional Research Institute of Unani Medicine (RRIUM), Naseem Bagh Campus, University of Kashmir, Srinagar, India; 2 Department of Pharmaceutical Sciences, University of Kashmir, Srinagar, India; 3 Centre of Research for Development (CORD), University of Kashmir, Srinagar, India; 4 Department of Microbiology, Noakhali Science and Technology University, Noakhali, Bangladesh; 5 Department of Clinical Laboratory, College of Applied Medical Sciences, King Khalid University, Abha, Saudi Arabia; 6 Department of Chemistry, University of Kashmir, Srinagar, India; 7 Department of Zoology, Model Degree College, Charar-i-Sharief, India; 8 Department of Biotechnology, PSG College of Technology, Coimbatore, India; 9 Department of Bio-Resources, School of Biological Sciences, University of Kashmir, Srinagar, India

**Keywords:** antioxidant activity, anxiety disorders, anxiolytic effect, *Bunium persicum*, fatty acids, molecular docking, molecular dynamics simulation

## Abstract

**Ethnopharmacological Relevance:**

*Bunium persicum* is a valued traditional Persian medicinal spice used as a calming and anxiolytic agent. However, the bioactive metabolites responsible for its antioxidant and anxiolytic effects, and their underlying mechanisms, remain largely unexplored.

**Aim of the Study:**

This study evaluated the antioxidant and anxiolytic potential of hydroalcoholic extracts and solvent fractions of *B. persicum* fruits through bioactivity-guided isolation, supported by *in-vivo* and *in silico* validation to mechanistically confirm its traditional use in anxiety disorders.

**Materials and Methods:**

Fruits were extracted with 70% hydroalcoholic solvent and successively fractionated with solvents of increasing polarity. Antioxidant activity was assessed via total phenolic content (TPC), total flavonoid content (TFC), DPPH radical scavenging, and reducing power assays. The most active ethyl acetate fraction underwent bioactivity-guided isolation, yielding three fatty acids (myristic, palmitic, and stearic acid) characterized by spectroscopic methods. Anxiolytic effects of the isolates were tested in Swiss albino mice using elevated plus maze (EPM) and light–dark arena (LDA) tests. Acute oral toxicity was evaluated per OECD guidelines (2000 mg/kg). In-silico studies included molecular docking against MAO, GABA(A) β3 subunit, COMT, and SERT, followed by 200 ns MD simulations to assess binding stability.

**Results:**

The ethyl acetate fraction showed the highest antioxidant activity (TPC: 337.408 ± mg GAE/g; TFC: 286.665 ± mg RU/g) with superior DPPH scavenging and reducing power. Stearic acid exhibited significant anxiolytic-like effects in both EPM and LDA tests (P < 0.01–0.001 vs. control), comparable to diazepam, without sedation or motor impairment. No acute toxicity was observed at 2000 mg/kg. Docking revealed strong binding affinities (ΔG = −5.3 to −7.6 kcal/mol) driven by hydrogen bonds and hydrophobic interactions. MD simulations confirmed complex stability over 200 ns with favourable RMSD, RMSF, and persistent interactions.

**Conclusion:**

This study provides the first mechanistic evidence supporting the traditional Persian use of *B. persicum* as a natural anxiolytic. Stearic acid, isolated via bioactivity-guided fractionation, emerges as a promising multi-target antioxidant and anxiolytic scaffold, validating its ethnomedicinal relevance and potential for safer plant-derived therapeutics against anxiety disorders.

## Graphical Abstract

**Figure:**
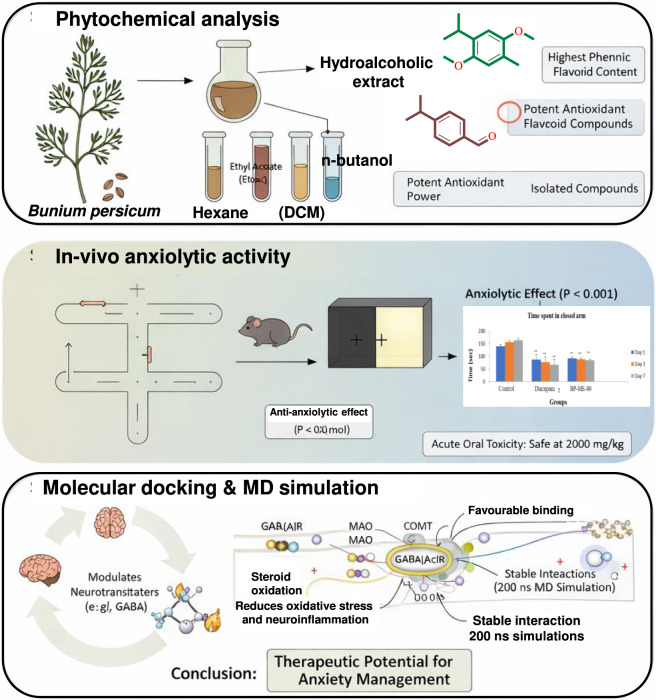

## 1 Introduction

Anxiety disorders represent the most prevalent mental health conditions globally, affecting millions and leading to substantial morbidity through reduced quality of life and frequent comorbidity with depression and other disorders (Javaid et al., 2023; Kumar et al., 2024). Growing evidence links anxiety pathophysiology to oxidative stress an imbalance between reactive oxygen species (ROS) production and antioxidant defenses that damages neural cells along with downstream neuroinflammation, dysregulation of key neurotransmitter systems (e.g., GABAergic and serotonergic), and impaired neurogenesis (Houldsworth, 2024; Nunes et al., 2025). These interconnected mechanisms underscore the therapeutic potential of natural compounds with potent antioxidant and anti-inflammatory properties as safer alternatives or adjuncts to conventional treatments like benzodiazepines and selective serotonin reuptake inhibitors, which carry risks of dependence, tolerance, and adverse effects (Hassamal, 2023; Kenda et al., 2022).

*Bunium persicum* (Boiss.) B. Fedtsch. (Apiaceae), commonly known as black cumin or Kala Zeera, is a perennial aromatic herb native to Iran, Jammu and Kashmir (India), and Central Asia. Traditionally used in folk medicine as a carminative, antispasmodic, digestive aid, and calming remedy, its fruits (seeds) serve both culinary and medicinal purposes (Sofi et al., 2009; Majidi et al., 2020). Phytochemical profiling reveals a rich composition dominated by essential oils (e.g., γ-terpinene, cuminaldehyde, p-cymene, limonene), phenolics, flavonoids, and fatty acids (Mehrabadi and Zarshenas, 2021). Extracts and essential oils exhibit robust antioxidant activity in assays such as DPPH radical scavenging, β-carotene bleaching, and reducing power (Nickavar et al., 2014), alongside documented anticonvulsant effects (Mandegary et al., 2012). While related Apiaceae species show anxiolytic promise, direct studies on *Bunium persicum* anxiolytic activity remain scarce.

Recent findings suggest that long-chain saturated fatty acids such as myristic (C14:0), palmitic (C16:0), and stearic (C18:0) acids isolated from plant sources may contribute to antioxidant, neuroprotective, and anxiety-modulating effects via ROS scavenging and anti-inflammatory pathways (Hajhashemi et al., 2011; RC and Kakkalameli, 2025). Notably, preclinical models indicate differential profiles among these acids: myristic acid displays anxiolytic-like effects in elevated plus maze tests at low doses, palmitic acid can induce anxiogenic behavior with elevated amygdala serotonin turnover, and stearic acid appears largely neutral or supportive in oxidative stress contexts (Contreras et al., 2014; Moon et al., 2014; Wang et al., 2006; García-Ríos et al., 2013). Given *B. persicum*’*s* established antioxidant profile, particularly in its ethyl acetate fraction enriched in such metabolites, and the plant’s traditional use for calming effects, further investigation into its anxiolytic potential is warranted.

While prior research on *B. persicum* has extensively documented the antioxidant, anticonvulsant, anti-inflammatory, analgesic, and antimicrobial properties of its essential oils and crude extracts, direct evaluation of anxiolytic activity especially through bioactivity-guided isolation and purification of individual metabolites remains limited. No studies have isolated myristic, palmitic, and stearic acids from its ethyl acetate fraction or assessed their anxiolytic potential using combined *in-vivo* behavioral paradigms and multi-target computational validation. The present study addresses this critical gap by evaluating the antioxidant capacity (total phenolic/flavonoid content, DPPH scavenging, reducing power) of hydroalcoholic extracts and solvent fractions of *B. persicum* fruits, isolating and characterizing these three fatty acids from the most active ethyl acetate fraction, and investigating anxiolytic effects via elevated plus maze and light–dark arena tests in rodents. Mechanistic insights are provided through molecular docking and 200 ns molecular dynamics simulations against key anxiety-related targets (MAO-A, GABA_A receptor β3 subunit, COMT, and SERT). This integrated approach bridges ethnopharmacological traditions of *B. persicum* as a natural calming agent in Persian and regional folk medicine with contemporary neuropharmacological evidence, offering the first comprehensive exploration of its fatty acid components as potential multi-target natural antioxidants and anxiolytics.

## 2 Materials and methods

### 2.1 Drugs and chemicals

All chemicals used in this study were of analytical grade and procured from SRL Chemicals and Merck, India. Silica gel (60–120 mesh and 200–400 mesh) from Merck, India, was used for column chromatography, while pre-coated thin-layer chromatography (TLC) plates from the same supplier were employed for monitoring reactions. Anhydrous sodium sulfate was used as the drying agent for organic extracts. All weighings were carried out on a single-pan electronic balance. Melting points of the isolated metabolites were determined using a Perfit melting point apparatus (uncorrected). ^1^H NMR spectra were recorded on a Bruker Spectrospin spectrometer operating at 400 MHz, and ^13^C NMR spectra at 100 MHz, using TFA or DMSO-d_6_ as solvents and tetramethylsilane (TMS) as the internal standard. Mass spectra were obtained by electron impact (EI) ionization at 70 eV. The standard drug, diazepam, was obtained from Ranbaxy Laboratories, India, and carboxymethylcellulose (CMC) was purchased from CDH Laboratory, New Delhi, India.

### 2.2 Plant material, collection, authentication and preparation of specimen

Fresh fruits of the *B. persicum* were collected from Gurez, Bandipora, Jammu and Kashmir, India. The fruits were identified and authenticated by Mr. Akhtar H. Malik, Curator, Centre for Biodiversity and Taxonomy (CBT), Department of Botany, University of Kashmir, and a voucher specimen (No. 2710-KASH) was deposited. The plant material was subsequently air-dried and ground into a fine powder using a mixer grinder. This powder was then subjected to hydroalcoholic extraction, followed by fractionation using various organic solvents and evaluation of pharmacological parameters.

### 2.3 Plant extraction

The dried fruits of *B*. *persicum* (2 kg) were extracted following standard procedures. The fresh fruits were initially shade-dried and then powdered using a mechanical grinder. The powdered material was subjected to exhaustive extraction with a hydroalcoholic solvent mixture (ethanol: water, 80:20 v/v) using a Soxhlet apparatus for 72 h. The resulting extract was filtered while hot through Whatman filter paper, concentrated under reduced pressure in a rotary evaporator, and finally dried in a desiccator. This process yielded approximately 200 g of a dark brown crude hydroalcoholic extract. The extract was stored at 2 °C–4 °C until further analysis.

### 2.4 Fractionation

The hydroalcoholic extract obtained from the dried fruits of *B. persicum* was fractionated by suspending it in water and successively partitioning it with organic solvents of increasing polarity *viz*., hexane, dichloromethane (DCM), ethyl acetate, and n-butanol using a separating funnel. Each fraction was concentrated to dryness by evaporating the respective solvent under reduced pressure using a rotary evaporator and then stored at 4 °C.

### 2.5 Experimental animals

The animal studies were approved by the Institutional Animal Ethics Committee of the Indian Institute of Integrative Medicine (IIIM), Jammu, Jammu & Kashmir, India, a facility registered with the Committee for the Purpose of Control and Supervision of Experiments on Animals (CPCSEA), Government of India (Registration No. 801/GO/Re/S/2003/CPCSEA). Swiss albino mice of either sex (25–30 g) were procured from the Animal House of IIIM Jammu. All experiments were conducted in strict accordance with the institutional guidelines, CPCSEA regulations, and the national legislation for the care and use of laboratory animals. The Swiss albino mice were housed in polypropylene cages and maintained under standard environmental conditions (temperature: 25 °C ± 2 °C; relative humidity: 45%–55%; 12:12 h light: dark cycle), with free access to food and water *ad libitum*. All experiments were carried out during the light period (08:00–18:00 h).

### 2.6 Acute oral toxicity study

The acute oral toxicity study was conducted in accordance with OECD Guideline 425 (Up-and-Down Procedure). Swiss albino mice were used as the animal model.

Initially, a single mouse was administered a dose of 2000 mg/kg body weight (p.o.) of the hydroalcoholic extract of *B. persicum* fruits and observed for 24 h. As the animal survived, four additional mice were similarly dosed at 2000 mg/kg body weight (p.o.) and monitored for 24 h. All five animals survived the treatment period, indicating that the median lethal dose (LD_50_) of the extract is greater than 2000 mg/kg body weight. Based on these results, doses of 200 mg/kg body weight (1/10th of 2000 mg/kg) and 400 mg/kg body weight (1/5th of 2000 mg/kg) were selected for subsequent experimental evaluations.

### 2.7 Phytochemical analysis

#### 2.7.1 Total phenol content

The total phenolic content of hexane, dichloromethane (DCM), ethyl acetate, and butanol fractions of the hydroalcoholic extract from the fruits of *B. persicum* was estimated by the Folin-Ciocalteu method, as described by (Idris et al., 2017) with some modifications and gallic acid as a standard phenolic metabolite. In brief, 0.5 mL of each fraction solution (1 mg/mL) was combined with 2.5 mL of 10% Folin-Ciocalteu reagent and 2 mL of 2% (w/v) Na_2_CO_3_ solution. The mixture was left at 45 °C with agitation for 15 min. The absorbance was then read at 765 nm using a UV spectrophotometer. The result was calculated as milligrams of gallic acid equivalents (GAE) per Gram of fraction, using a standard curve prepared with gallic acid (0–0.5 mg/mL in distilled water).

#### 2.7.2 Total flavonoid content

The total flavonoid content was determined using the AlCl_3_ colorimetric assay (Ohikhena et al., 2018) with some modifications. In summary, a mixture of 1 mL sample solution (1 mg/mL), 3 mL methanol, 0.2 mL 10% AlCl_3_ solution, 0.2 mL 1 M potassium acetate, and 5.6 mL distilled water was incubated at room temperature for 30 min and then measured for absorbance at 420 nm. The flavonoid content was quantified using a rutin standard curve (0–0.8 mg/mL).

#### 2.7.3 DPPH radical scavenging assay

DPPH assay was performed following the methodology used by (Nickavar et al., 2008) with slight modification. Briefly, 1 mL of 0.135 mM DPPH made in methanol was mixed with 1.0 mL of hexane, DCM, ethylacetate and butanol fractions of hydroalcoholic extracts of fruits of *B*. *persicum* ranging from 0.2 to 0.8 mg/mL. The reaction mixture of the extracts was shaken and incubated for 30 min, left in dark at room temperature and the absorbance was read at 517 nm spectrophotometrically. The scavenging effect of the plant extracts was measured as the decrease in the absorbance of DPPH and calculated by applying the following equation (Braca et al., 2002; Aiyegoro and Okoh, 2019).

$$\text{DPPH Scavenging Activity }\left(\%\right)=\left[\left({\text{Abs}}_{\text{control}}-{\text{Abs}}_{\text{sample}}\right)\right]/\left[\left({\text{Abs}}_{\text{control}}\right)\right]x\;100$$

Where Abs_control_ is the absorbance of DPPH + Methanol Abs_sample_ is the absorbance of DPPH radical + Sample (i.e., extract or standard).

#### 2.7.4 Reducing power assay

The total reducing power of the plant extracts was determined by assessing their ability to reduce ferric ions (Fe^3+^), based on the method described by (Moein et al., 2008) with minor modifications. Briefly, 1.0 mL of each extract (dissolved in distilled water) was mixed with 2.5 mL of 0.2 M phosphate buffer (pH 6.6) and 2.5 mL of 1% (w/v) potassium ferricyanide [K_3_Fe (CN)_6_]. The mixture was incubated in a water bath at 50 °C for 20 min. Subsequently, 2.5 mL of 10% (w/v) trichloroacetic acid (TCA) was added, and the mixture was centrifuged at 3,000 rpm for 10 min when necessary. The upper layer (2.5 mL) was then collected, mixed with 2.5 mL of distilled water and 0.5 mL of freshly prepared 0.1% (w/v) ferric chloride (FeCl_3_). The absorbance was measured at 700 nm against a blank.

### 2.8 Phytochemistry of *B. persicum*

#### 2.8.1 Column chromatography of ethylacetate fraction of *B. persicum*

**FIGURE 1:**
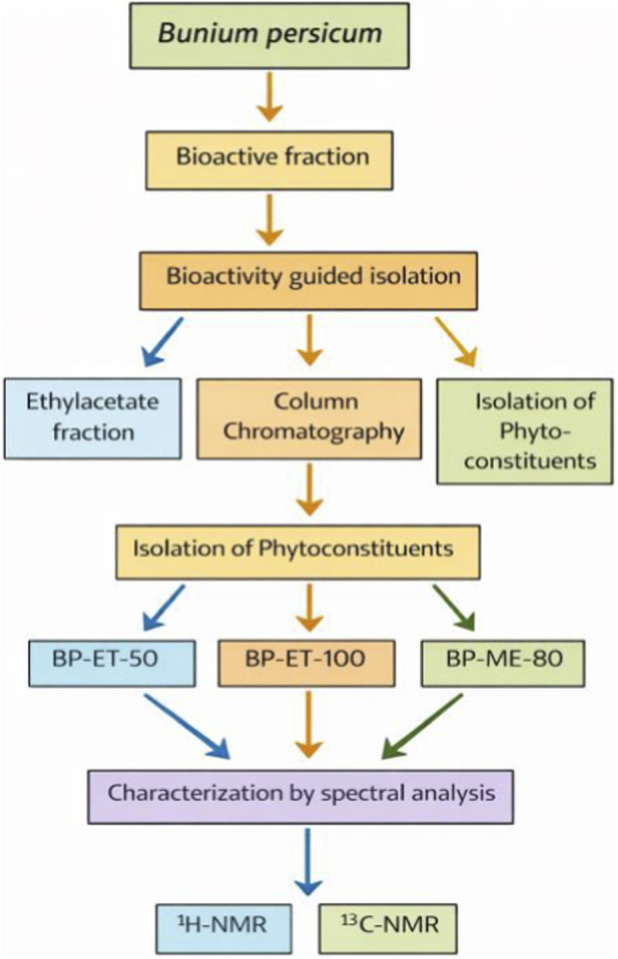
Flowchart illustrating the bioactivity-guided isolation and structural characterization of phytoconstituents from *Bunium persicum*.

Column chromatography was performed using silica gel (200–400 mesh). A clean, dry column was clamped vertically, washed with water, rinsed with acetone, and dried. The ethyl acetate fraction was dissolved in minimal methanol, adsorbed onto silica gel (60–120 mesh), and dried to a free-flowing powder. This slurry was loaded onto the column top. Elution was carried out with hexane–ethyl acetate gradients (100:0 to 0:100 v/v) followed by ethyl acetate–methanol gradients (98:2 to 0:100 v/v). Fractions (50 mL each) were collected, monitored by TLC, and similar fractions pooled based on identical Rf values. Chromatograms were visualized under UV light and after derivatization with anisaldehyde–sulfuric acid reagent. Pooled fractions were concentrated, dried, weighed, and characterized (Figure 1).

#### 2.8.2 Characterization of isolated metabolites

The isolated metabolites were characterized to determine the structure by instrumental spectral analysis such as ^1^HNMR and ^13^CNMR.

### 2.9 Animal models used for screening of anxiolytic activity of the isolated metabolites

#### 2.9.1 Elevated plus maze

The elevated plus maze consisted of two open arms (16 × 5 cm) and two closed arms (16 × 5 × 12 cm) arranged opposite each other, elevated 25 cm above the ground. Mice were administered test substances orally and, 45 min later, placed individually in the center facing an open arm. Behavior was recorded for 5 min during the dark phase (9:00–16:00 h). Parameters measured included: (i) number of entries into open and closed arms, and (ii) time spent in open and closed arms. Animals were allowed to socialize between tests, and care was taken to minimize external stimuli (Bourin et al., 2007).

#### 2.9.2 Light & dark arena model

The light/dark apparatus was a wooden box with a brightly illuminated white chamber (30 × 30 × 35 cm) and a dark black chamber (20 × 30 × 35 cm) connected by a small opening (7.5 × 5 cm). The light chamber was illuminated by a 100 W bulb positioned 17 cm above. Forty-five minutes post-oral administration, each mouse was placed in the center of the light chamber and observed for 5 min. Parameters recorded were: (i) time spent in each chamber, and (ii) number of transitions between chambers (Bourin and Hascoët, 2003).

#### 2.9.3 Hole board apparatus model

The hole board apparatus was a Perspex box (60 × 60 × 35 cm) with four equidistant holes (3 cm diameter) in the floor, illuminated by a 100 W bulb. The floor was marked with lines dividing it into 9 cm squares and elevated 25 cm above the ground. Forty-five minutes after oral administration, each mouse was placed in the center and allowed to explore freely for 5 min. Parameters measured included: (i) number of squares crossed (locomotor activity), (ii) number and duration of head dips, and (iii) latency to first head dip (Kong et al., 2006; Casarrubea et al., 2023; Rodriguez Echandia et al., 1987; Takeda et al., 1998; Vinadé et al., 2003).

#### 2.9.4 Modified forced swimming bath test

The apparatus consisted of a Plexiglas tank (20 × 8 × 18 cm) containing a central water wheel (Plexiglas shaft: 3 cm diameter × 6 cm length with six 0.5 cm-wide paddles). The tank was filled with water (25 °C) to 9 cm depth, allowing paddles to contact the surface. Wheel rotations were counted when triggered by weights >5 g (Porsolt et al., 1979).

### 2.10 Computational studies

#### 2.10.1 Preparation of protein and ligands

Crystal structures of anxiety-related target proteins such as Human Monoamine Oxidase (MAO) (PDB ID: 2Z5X), Human gamma-aminobutyric acid receptor, the GABA(A)R-beta3 homopentamer (PDB ID: 4COF), Catechol-O-methyltransferase (COMT) (PDB ID: 4XUC) and Serotonin transporter (PDB ID: 7LIA) were retrieved in PDB format from the RCSB Protein Data Bank (Rose et al., 2016). Proteins were prepared using the Protein Preparation Wizard in Maestro v12.5.139 (Schrödinger, LLC) (Shaw et al., 2010). For 4COF, only chain A was retained, with chains B–E removed. Heteroatoms, water molecules, and co-crystallized ligands were deleted from all structures. Preprocessing involved adding missing hydrogen atoms, verifying sequences, and optimizing side chains using default settings. Final minimization was performed with the OPLS-3e force field (Chow et al., 2017). Stearic acid, palmitic acid, and myristic acid were isolated from *B. persicum* and identified by column chromatography. Their 3D structures were downloaded in SDF format from PubChem (Kim et al., 2023) using PubChem IDs: 5,281 (stearic acid), 985 (palmitic acid), and 11,005 (myristic acid). Ligands were prepared using LigPrep in Maestro, with Epik v5.3 for generating appropriate protonation and tautomeric states at physiological pH. Optimized geometries were energy-minimized using the OPLS-3e force field to ensure compatibility with the prepared proteins.

#### 2.10.2 Molecular docking analysis

Molecular docking was performed to evaluate the binding affinities and interaction profiles of the isolated phytochemicals like stearic acid, palmitic acid, and myristic acid against anxiety-related target proteins (PDB IDs: 2Z5X, 4COF, 4XUC, and 7LIA). Docking was conducted using the Glide v8.8 module in Maestro v12.5.139 (Schrödinger Suite) with the OPLS-3e force field in standard precision (SP) mode. Receptor grids were centered on the active sites with coordinates: 2Z5X (X = 40.733, Y = 26.871, Z = −14.816), 4COF (X = −20.674, Y = −19.579, Z = 128.198), 4XUC (X = −2.890, Y = 5.863, Z = −16.622), and 7LIA (X = 128.917, Y = 127.478, Z = 137.770). Docking scores indicated binding strengths, and interactions (hydrogen bonds, hydrophobic contacts) were visualized and analyzed using Maestro Viewer.

#### 2.10.3 MD simulation analysis

The stability of top-ranked protein–ligand complexes was assessed via 200 ns MD simulations using Desmond (Schrödinger Suite) (Shaw et al., 2010). Complexes from docking served as starting structures. The system was built with the SPC solvent model in an orthorhombic box (10 Å buffer), neutralized, and adjusted to 0.15 M NaCl to mimic physiological conditions. Simulations were run in the NPT ensemble (300 K, 1.01325 bar) using Nose–Hoover thermostat and isotropic scaling. Energy minimization and equilibration employed the OPLS-3e force field (Chow et al., 2017). Trajectories were saved every 200 ps. post-simulation analyses included RMSD (protein and ligand), RMSF, Rg, SASA, protein–ligand contacts, and hydrogen bonds. Conformational dynamics were further explored through Principal Component Analysis (PCA) and Dynamic Cross-Correlation Matrix (DCCM) using the Bio3D package in R with custom scripts.

### 2.11 Statistical analysis

All data are expressed as Mean ± SEM and statistical analysis was carried out using one way analysis of variance (ANOVA) followed by Dunnet’s Multiple Comparison Test *P < 0.05, **P < 0.01, ***P < 0.001). P value more than 0.05 was considered as statistically not significant.

## 3 Results

### 3.1 Macroscopic and organoleptic characteristics

**FIGURE 2:**
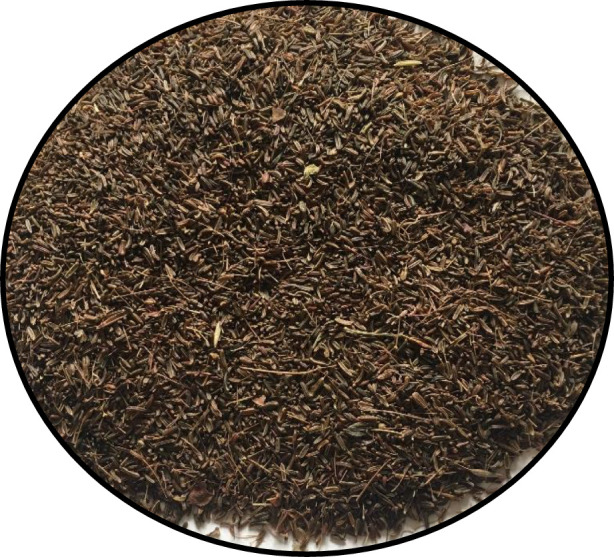
Macroscopic view of *B. persicum* fruits.

The fruits of *B. persicum* are black-brownish in colour, crescent-shaped, approximately 4.7 mm long and 1.6 mm wide, with an astringent taste and aromatic odour (Figure 2).

### 3.2 Powder microscopy of fruits of *B. persicum*

#### 3.2.1 Powder microscopy and diagnostic characters

**FIGURE 3:**
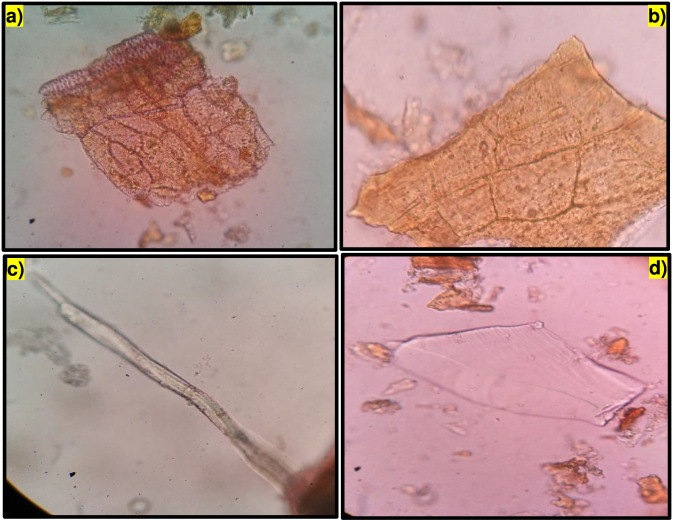
Microscopic structures of *B. persicum* fruit powder **(a)** Group of sclereids from mesocarp; **(b)** Fragments of vittae; **(c)** Thin-walled fibres; **(d)** Prismatic calcium oxalate crystals.

Powder microscopy revealed diagnostic features including large groups of sclereids from the mesocarp (rectangular to subrectangular, moderately to heavily thickened walls with regularly spaced pits, often associated with thin-walled unlignified parenchyma). Brown vittae fragments composed of thin-walled polygonal cells, occasional fibro-vascular tissue with thin-walled fibres, and endosperm containing prismatic calcium oxalate crystals were prominent (Figure 3).

### 3.3 Physicochemical constants

The physicochemical constants of *B. persicum* fruits provide essential quality parameters for standardization and authentication of the crude drug. As presented in Table 1, the total ash value was 7.4%, acid-insoluble ash 2.15%, and sulphated ash 0.4%, indicating low levels of inorganic impurities and silica-containing materials. Extractive values demonstrated good solubility in polar solvents: the hot aqueous extractive value reached 31.8% (w/w), hot ethanolic 17.5%, cold aqueous 15.6%, and cold ethanolic 8.0%, reflecting the abundance of polar bioactive constituents. Loss on drying was 6.2%, suggesting moderate moisture content suitable for storage stability. The pH values of 1% and 10% aqueous solutions were 6.5 and 6.1, respectively, indicating a slightly acidic nature of the fruit powder. The swelling index was recorded as 1, consistent with limited mucilage content. Foreign organic matter was negligible at 0.016% (Table 1), confirming high purity of the collected material.

**TABLE 1:** Physicochemical constants of fruit part of *B. persicum*

Parameter	Value
Total ash	7.4%
Acid-insoluble ash	2.15%
Sulphated ash	0.4%
Ethanol extractive (hot)	17.5%
Aqueous extractive (hot)	31.8%
Ethanol extractive (cold)	8.0%
Aqueous extractive (cold)	15.6%
Foreign organic matter	0.016%
Loss on drying	6.2%
pH (1% solution)	6.5
pH (10% solution)	6.1
Swelling index	1

### 3.4 Microbial load

The microbial load analysis of *B. persicum* fruits was conducted to assess microbiological safety and quality as per standard pharmacopoeial guidelines for crude botanical drugs. As detailed in Table 2, the total bacterial count was 100 CFU/g, which is well below the WHO-recommended limit of NMT 1000 CFU/g for many crude plant materials and botanical drug preparations not subjected to sterilization. Yeast and mould counts were nil (absent), complying with the typical WHO limit of NMT 100 CFU/g. Specific pathogenic organisms tested were negative: *Escherichia coli*, *Staphylococcus aureus*, and *Salmonella* spp. were absent, meeting WHO and pharmacopoeial requirements for absence in 1 g or specified quantities.

**TABLE 2:** Determination of Microbial load of *B. persicum*

Parameter	Value obtained	WHO limit
Total bacterial count	100 CFU/g	NMT 1000 CFU/g
Yeast and moulds	Nil	NMT 100 CFU/g
*Escherichia coli*	Negative	Absent
*Staphylococcus aureus*	Negative	Absent
*Salmonella*	Negative	Absent

CFU: colony forming unit.

### 3.5 Heavy metal and mineral content

The heavy metal and mineral content of *B. persicum* fruits was analyzed using standard analytical techniques (e.g., atomic absorption spectroscopy or ICP-based methods) to evaluate safety and compliance with regulatory standards for crude botanical drugs. As shown in Table 3, heavy metal levels were extremely low: chromium was 0 ppm, nickel 0.117 ppm, cadmium 0.008 ppm, and lead 0.307 ppm. All values are well below the permissible limits commonly referenced in WHO guidelines and pharmacopoeial standards for raw medicinal plants (e.g., cadmium NMT 0.3 ppm, lead NMT 1.0–5.0 ppm depending on source, chromium NMT 2.0 ppm in some references, nickel NMT 1.63 ppm), indicating negligible risk of heavy metal toxicity. Table 4 presents the mineral content: copper 0.090 ppm (NMT 3.00 ppm permissible), zinc 0.364 ppm (NMT 27.4 ppm), manganese 0.174 ppm (no specific WHO limit), magnesium 38.85 ppm (no regulatory limit), cobalt 0.056 ppm (no limit), and iron 0.242 ppm (NMT 20.0 ppm in some guidelines). These essential minerals are present at trace to moderate levels, consistent with natural plant composition and far below any established upper thresholds where applicable.

**TABLE 3:** Heavy metal analysis of fruit part of *B. persicum*

Heavy metal	Content (ppm)	Permissible limit (ppm)
Chromium	0	NMT 2.0
Nickel	0.117	NMT 1.63
Cadmium	0.008	NMT 0.3
Lead	0.307	NMT 1.0

**TABLE 4:** Mineral content of fruit part of *B. persicum*

Mineral	Content (ppm)	Permissible limit (ppm)
Copper	0.090	NMT 3.00
Zinc	0.364	NMT 27.4
Manganese	0.174	No WHO limit
Magnesium	38.85	No WHO limit
Cobalt	0.056	No WHO limit
Iron	0.242	NMT 20.0

### 3.6 Yield of extraction

The yields of the fractions were as follows: hexane fraction (29 g), DCM fraction (13 g), ethyl acetate fraction (14 g), and butanol fraction (65 g). These fractions were subsequently subjected to phytochemical screening as well as evaluation for antioxidant and antianxiety activities. Metabolite isolation was performed from the most bioactive fraction. Among the fractions, the ethyl acetate fraction exhibited the highest antioxidant potential and was identified as the most bioactive fraction.

### 3.7 Phytochemical analysis

#### 3.7.1 Total phenolic content

**FIGURE 4:**
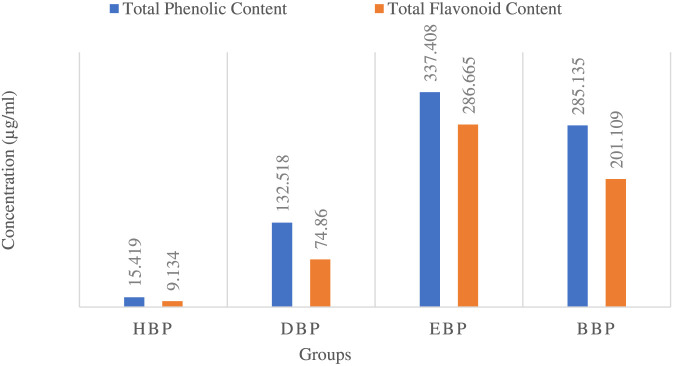
Illustrates the total phenolic content (TPC) and flavonoid (TFC) content of hexane (HBP), dichloromethane (DBP), ethyl acetate (EBP), and butanol (BBP) fractions of *B. persicum* fruits, expressed as mg GAE/g extract.

The total phenolic content (TPC) of the hexane (HBP), dichloromethane (DBP), ethyl acetate (EBP), and butanol (BBP) fractions obtained from *B. persicum* fruits was determined using the Folin–Ciocalteu method (Pérez et al., 2023). Results were expressed as milligrams of gallic acid equivalents (GAE) per Gram of extract (mg GAE/g). A standard calibration curve was prepared using gallic acid (3.33–106.66 μg/mL), yielding the equation y = 0.0143x + 0.0576 (R^2^ = 0.9973), where y is absorbance at 765 nm and x is gallic acid concentration (µg/mL). Absorbance values of the fractions (200 µL of 1 mg/mL extract) at 765 nm were 0.071 (HBP), 0.183 (DBP), 0.379 (EBP), and 0.329 (BBP) (Table 5). The TPC varied significantly among the fractions, with the ethyl acetate fraction (EBP) exhibiting the highest content (337.408 mg GAE/g), followed by the butanol (BBP; 285.135 mg GAE/g), dichloromethane (DBP; 132.518 mg GAE/g), and hexane (HBP; 15.419 mg GAE/g) fractions (Table 5; Figure 4). These results indicate that phenolic metabolites were preferentially extracted in mid-polarity solvents (ethyl acetate and butanol), while the non-polar hexane fraction contained the lowest levels.

**TABLE 5:** Absorbance at 765 nm and total phenolic content of *B. persicum* fruit fractions.

Sample	Volume (µL)	Concentration in assay (µg/mL)	Absorbance (765 nm)	Total phenolic content (mg GAE/g extract)
Gallic acid (GAE)	​	​	​	​
	10	3.33	0.068	–
	20	6.66	0.152	–
	40	13.33	0.249	–
	80	26.66	0.458	–
	160	53.33	0.868	–
	320	106.66	1.558	–
*B. persicum* fractions (1 mg/mL)				
Hexane (HBP)	200	66.67	0.071	15.419
Dichloromethane (DBP)	200	66.67	0.183	132.518
Ethyl acetate (EBP)	200	66.67	0.379	**337.408**
Butanol (BBP)	200	66.67	0.329	285.135

#### 3.7.2 Total flavonoid content

The total flavonoid content (TFC) of *B. persicum* fruit fractions of the hexane (HBP), dichloromethane (DBP), ethyl acetate (EBP), and butanol (BBP) was determined by aluminum chloride colorimetry and calculated as mg rutin equivalents (RU/g) of extract. With a rutin standard (3.33–106.66 μg/mL), the calibration curve was y = 0.0658x + 0.0143 (R^2^ = 0.997), where y is the absorbance at 415 nm and x is the rutin concentration in µg/mL. Absorbance of 200 µL of 1 mg/mL extract at 415 nm: HBP 0.073, DBP 0.0136, EBP 0.339, BBP 0.257 (Table 6; Figure 4). TFC was highly variable among fractions, with EBP having the highest content of 286.665 mg RU/g, BBP 201.109 mg RU/g, DBP 74.860 mg RU/g, and HBP 9.134 mg RU/g (Table 6). This pattern is consistent with the total phenolic content, suggesting that flavonoids are best extracted by mid-polar solvents (ethyl acetate and butanol), with minimal levels in the non-polar hexane fraction.

**TABLE 6:** Absorbance at 415 nm and total flavonoid content of *B. persicum* fruit fractions.

Sample	Volume (µL)	Concentration in assay (µg/mL)	Absorbance (415 nm)	Total flavonoid content (mg RU/g extract)
Rutin standard (RU)	​	​	​	​
	–	3.33	0.072	–
	–	6.66	0.162	–
	–	13.33	0.259	–
	–	26.66	0.468	–
	–	53.33	0.875	–
	–	106.66	1.568	–
*B. persicum* fractions (1 mg/mL initial)				
Hexane (HBP)	200	66.67	0.073	9.134
Dichloromethane (DBP)	200	66.67	0.136	74.860
Ethyl acetate (EBP)	200	66.67	0.339	**286.665**
Butanol (BBP)	200	66.67	0.257	201.109

#### 3.7.3 DPPH radical scavenging assay

**FIGURE 5:**
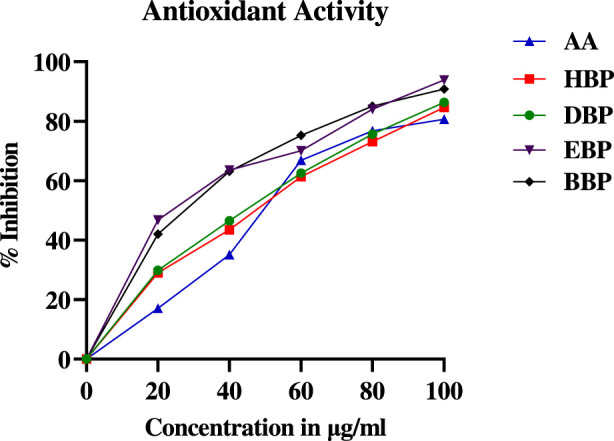
Graphical representation of DPPH radical scavenging capacity of Ascorbic acid, Hexane, DCM, Ethylacetate and Butanol fractions of *B. persicum*.

All fractions exhibited concentration-dependent DPPH radical scavenging capacity. The ethyl acetate (EBP) and butanol (BBP) fractions displayed the strongest scavenging potential, while the hexane (HBP) fraction showed the weakest activity. The standard ascorbic acid exhibited superior activity compared to all plant fractions (Figure 5; Supplementary Table S1). These findings indicate that the more polar fractions possess superior free radical scavenging capacity.

#### 3.7.4 Reducing power method

**FIGURE 6:**
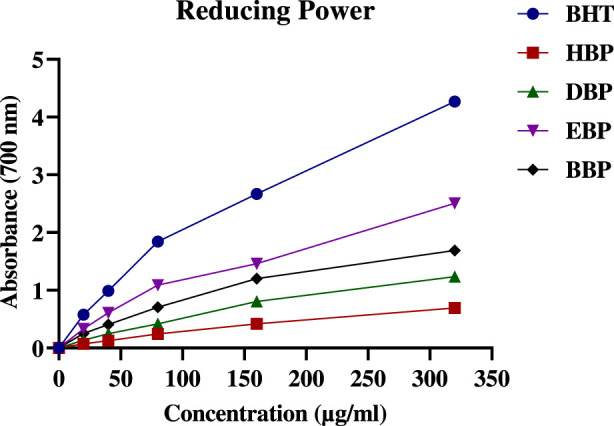
Graphically representation of reducing power activity of BHT, hexane, DCM, Ethylacetate and Butanol fractions of *B. persicum*.

The results revealed that with the increase in the concentration of the fractions there was an increase in their reducing power. The reducing power ability of various fractions of fruits of *B*. *persicum* are given below (Supplementary Table S2; Figure 6).

### 3.8 Column chromatography

#### 3.8.1 Isolation and characterization of pure metabolites from ethylacetate fraction of fruits of *B. persicum*

**FIGURE 7:**
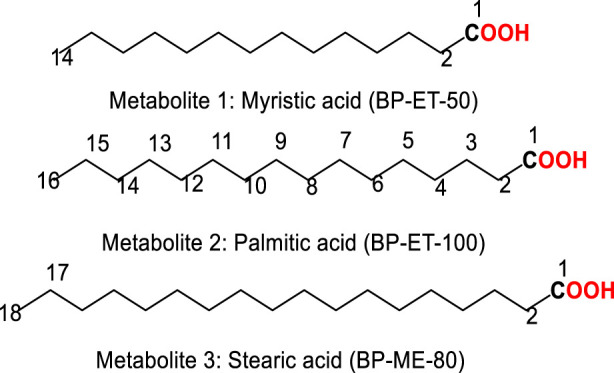
Structures of isolated metabolites from fruits of *B. persicum*.

The ethyl acetate fractions of the fruits of *B. persicum* extract were then subjected to a phytochemical investigation, which led to the isolation of three metabolites. The isolated metabolites were identified by using mass and NMR (^1^H, ^13^C, spectral data as: Myristic Acid (Tetradecanoic acid) (BP-ET-50) (1), Palmitic acid (BP-ET-100) (2), and Stearic acid (BP-ET-100) (BP-ME-80) (3) (Supplementary Figures S1a-c). Structures of these metabolites are shown in Figure 7. Chemical and physical properties of isolated metabolites were in agreement with reported data.

Myristic acid (BP-ET-50) was isolated from the hexane-ethylacetate subfractions as a brown colored solid mass. The metabolite showed no notable fluorescence under UV light. The mass spectrum of this metabolite based on its positive electrospray ionization mass spectrometry (+ve ESI-MS) displayed a pseudo-molecular ion at m/z 229 [M^+^H] ^+^ corresponding to the molecular formula C_14_H_28_O_2_. The structure of this metabolite was determined with the aid of one- and two-dimensional NMR spectral data including ^1^H NMR and ^13^C NMR, as well as comparison with reported long-chain fatty acid spectra. The ^1^H NMR spectrum (500 MHz, CDCl_3_) showed a broad singlet at δ 9.52 (^1^H) assignable to the carboxylic acid proton (COOH). A multiplet at δ 2.30–2.26 (2H) was assigned to the methylene protons adjacent to the carboxylic group (H-2). A multiplet at δ 1.65–1.61 (2H) corresponded to the β-methylene protons (H-3). The remaining chain methylene protons appeared as a large multiplet (broad signal) at δ 1.39–1.21 (20H, H-4 to H-13). Finally, a multiplet at δ 1.01–0.97 (3H) was assigned to the terminal methyl protons (H-14), appearing as a distorted triplet due to overlapping. The ^13^C NMR spectrum (125 MHz, common NMR solvents, consistent with CDCl_3_) exhibited signals at δ 177.13 (s, C-1, carboxylic carbon), 34.35 (s, C-2), 31.64 (s, C-12), 29.01 (d, J = 13.4 Hz, likely overlapping methylene carbons in the chain), 24.80 (s, C-3), 22.93 (s, C-13), and 14.01 (s, C-14, terminal methyl). The molecular formula indicated the presence of one double-bond equivalent, which was accounted for by the carboxylic group. In conclusion, these spectral data agree with those of Myristic acid (Majó et al., 2021).

Palmitic acid (BP-ET-100) was obtained as a yellow-colored powdered mass from the Hexane-Ethylacetate (0:100) eluent of the ethylacetate fraction of *B. persicum*. Its mass spectrum displayed a pseudo-molecular ion peak at 257 [M^+^H]^+^ corresponding to the molecular formula C_16_H_32_O_2_ of a long-chain fatty acid, with supportive fragments at m/z 154 [C_11_H_22_] ^+^ and m/z 103 [C_5_H_11_O_2_]^+^. The formula indicated the presence of one double-bond equivalent accounted for by the carboxylic group. The ^1^H NMR spectrum exhibited a singlet at δ 9.52 for the carboxylic proton, multiplets at δ 2.30–2.26 (H-2), 1.65–1.61 (H-3), a broad multiplet at δ 1.39–1.21 for the chain methylenes (H-4 to H-15), and a multiplet at δ 1.01–0.97 for the terminal methyl (H-16). The ^13^C NMR showed characteristic signals for the carboxylic carbon at δ 177.13 and alkyl chain carbons consistent with a saturated C16 fatty acid. These spectral data were in excellent agreement with literature values for palmitic acid (e.g., HMDB0000220, PubChem CID_985, and related databases). On the basis of the above discussion, the structure of BP-ET-100 was confirmed as Hexadecanoic acid (Palmitic acid). In conclusion, the isolation via column chromatography and spectral data (^1^H NMR, and ^13^C NMR) fully agree with those of palmitic acid.

Stearic acid (BP-ME-80) was obtained as a dark brown colored mass from the Ethylacetate: Methanol (20:80) eluent of the ethylacetate fraction of *B. persicum*. Its mass spectrum showed a pseudo-molecular ion peak at 283 [M-H]^+^ corresponding to the molecular formula C_18_H_36_O_2_ of a long-chain fatty acid. The formula indicated the presence of one double-bond equivalent accounted for by the carboxylic group. The ^1^H NMR spectrum exhibited a singlet at δ 9.52 for the carboxylic proton, multiplets at δ 2.30–2.26 (H-2), 1.65–1.61 (H-3), a broad multiplet at δ 1.39–1.21 for the chain methylenes (H-4 to H-17), and a multiplet at δ 1.01–0.97 for the terminal methyl (H-18). Consistent with earlier broad assignments, signals at δ 1.58 (adjacent methylenes) and δ 1.27 (bulk chain) with triplet at δ 0.87 for H-18 further support the structure. The ^13^C NMR showed characteristic signals for the carboxylic carbon at δ 177.13 and alkyl chain carbons consistent with a saturated C18 fatty acid. On the basis of the above discussion, the structure of BP-ME-80 was confirmed as Octadecanoic acid (Stearic acid).

### 3.9 *In-vitro* anxiolytic activity of isolated metabolite BP-ME-80

#### 3.9.1 Antianxiety activity of metabolite BP-ME-80 isolated from *B. persicum* in elevated plus maze

##### 3.9.1.1 First day study

In EPM open arm activity is sensitive to the anxiolytic effect of Diazepam. Diazepam as expected significantly increased the open arm entries (5.16 ± 0.307^*^) and time spent in open arms (166.5 ± 5.506^**^sec) as compared to the control group (4.16 ± 0.477) and (98.16 ± 11.167 s). A decrease in the time spent in the closed arms was observed in the mice receiving diazepam (86.5 ± 5.078^**^sec) compared to the control group (139.16 ± 8.348 s) indicating anxiolytic activity of diazepam. The metabolite BP-ME-80 isolated from *B*. *persicum* also increased open arm entries (6.16 ± 0.307^**^) and the time spent in open arms (159.5 ± 5.110^**^) compared to the control group indicating anxiolytic activity of the isolated metabolite.

##### 3.9.1.2 Repeated dose entry

**FIGURE 8:**
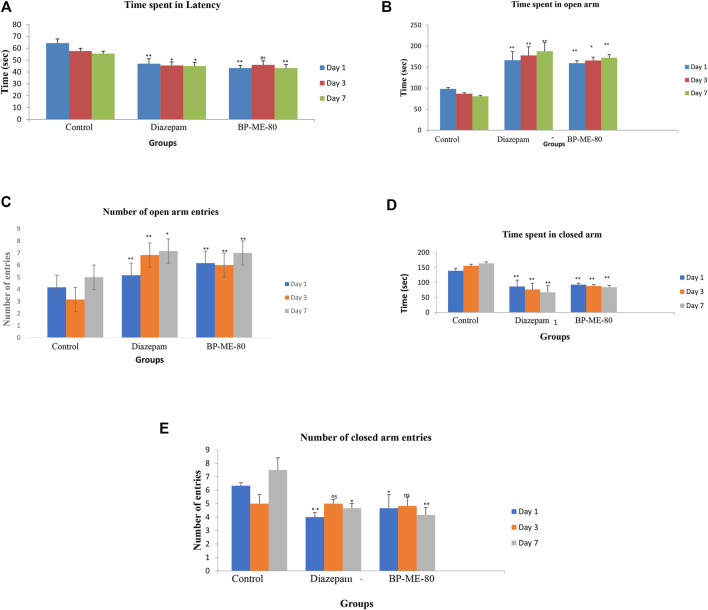
**(A)** Graphical representation of effect of metabolite BP-ME-80 isolated from *B persicum* on the time spent in latency. **(B)** Graphical representation of effect of metabolite BP-ME-80 isolated from *B persicum* on the time spent in open arm. **(C)** Graphical representation of effect of metabolite BP-ME-80 isolated from *B persicum* on the number of entries in open arm. **(D)** Graphical representation of effect of metabolite BP-ME-80 isolated from *B persicum* on the time spent in closed arm. **(E)** Graphical representation of effect of metabolite BP-ME-80 isolated from *B persicum* on the number of entries in closed arm.

Each group was retested on the 3^rd^ and 7^th^ day study after chronic treatment. Results revealed that compared to the 1^st^ day study, there was increase in the number of open arm entries and time spent in open arms on the 3^rd^ day in case of diazepam group (6.83 ± 0.600^**^; 177.83 ± 7.035^**^sec) and on the 7^th^ day of study there was marked increase in the open arm entries and time spent in the open arms (7.16 ± 0.401^*^; 187.83 ± 6.745^**^ sec), while as in case of Test group there was also increase in the number of open arm entries and time spent in open arms on the 3^rd^ day study: (6 ± 0.516^**^; 165.83 ± 4.615^*^) and on 7^th^ day study: (7 ± 0.577^*^; 172.5 ± 5.252^**^) compared to control group. The Test group receiving isolated metabolite BP-ME-80 had significant effect on the open arm entries and time spent in the open arms indicating anxiolytic activity of metabolite BP-ME-80 (Supplementary Table S3; Figures 8A–E).

### 3.10 Antianxiety activity of metabolite BP-ME-80 isolated from *B. persicum* in light and dark arena model

#### 3.10.1 First day study

In this model of anxiolytic study, results revealed that there was a significant increase in the time spent in the light box in case of Diazepam group (185.166 ± 5.186^**^) and Test group (171.16 ± 9.365^**^sec) compared to the control group (113.5 ± 6.228 s) indicating anxiolytic activity.

#### 3.10.2 Repeated dose study

**FIGURE 9:**
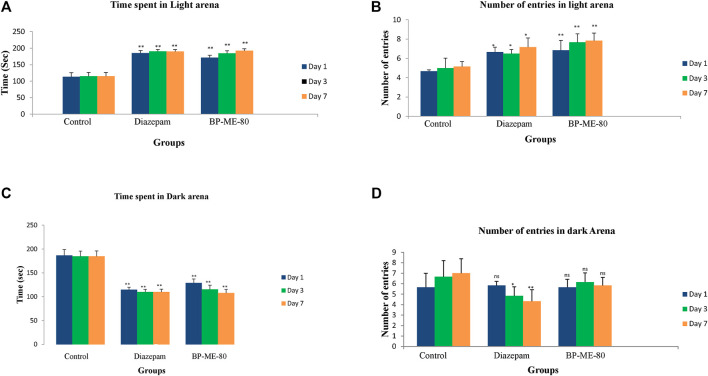
**(A)** Graphical representation of effect of metabolite BP-ME-80 isolated from *B. persicum* on the time spent in light arena. **(B)** Graphical representation of effect of metabolite BP-ME-80 isolated from *B. persicum* on the number of entries in light arena. **(C)** Graphical representation of effect of metabolite BP-ME-80 isolated from *B. persicum* on the time spent in dark arena. **(D)** Graphical representation of effect of metabolite BP-ME-80 isolated from *B. persicum* on the number of entries in dark arena.

Chronic study revealed that there was a significant increase in time spent in light box on the 3^rd^ day in case of (diazepam group: 190.16 ± 7.46^**^ sec, Test group: 184.5 ± 5.175^**^ sec) compared to 1^st^ day study and on the 7^th^ day study (diazepam group: 190 ± 6.563^**^sec, Test group: 192.16 ± 4.679^**^sec) compared to the control group. However, the overall number of transitions increased in the test group on chronic dosing indicating anxiolytic activity of the metabolite BP-ME-80 (Supplementary Table S4; Figures 9A–D).

### 3.11 Computational studies

#### 3.11.1 Molecular docking analysis

**FIGURE 10:**
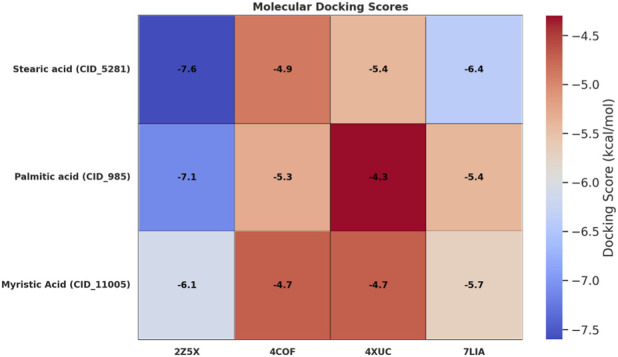
Heatmap illustrating the molecular docking scores (kcal/mol) of the three selected phytochemical metabolites (Stearic Acid, Palmitic Acid, and Myristic Acid) against four target proteins (2Z5X, 4COF, 4XUC, and 7LIA). The color gradient represents the binding affinities, with darker shades indicating stronger (more negative) interactions.

Molecular docking analysis revealed three isolated metabolites, namely, Stearic Acid, Palmitic Acid, and Myristic Acid CID_5281, CID_985, and CID_11005 as the top candidates due to their strong binding affinities against the target proteins. The most prominent interactions included CID_5281 with the 2Z5X protein (−7.6 kcal/mol) and 4XUC (−5.4 kcal/mol), CID_985 with 4COF (−5.3 kcal/mol), and CID_11005 with 7LIA (−5.7 kcal/mol) (Figure 10). The binding energies of these docked complexes reflect a favourable binding between target proteins and the selected metabolites, emphasizing the potential of the metabolites as lead molecules for downstream processes.

#### 3.11.2 Protein-ligand interaction analysis

**FIGURE 11:**
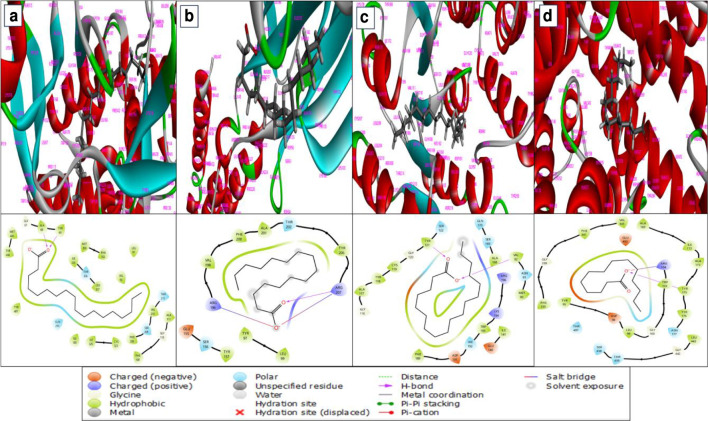
The figure represents the molecular interactions between the anxiety-related proteins (2Z5X, 4COF, 4XUC, and 7LIA) and the selected three metabolites (Stearic Acid, Palmitic Acid, and Myristic Acid). Here: 2Z5X_CID5281 **(a)**, 4COF_CID985 **(b)**, 4XUC_CID5281 **(c)**, and 7LIA_CID11005 **(d)**.

The interaction of the selected ligands with their target proteins was modeled using Maestro (Schrödinger suite). The interaction profile revealed non-covalent interactions such as hydrophobic, polar, and hydrogen bonds. During the docking process, stearic acid, palmitic acid, and myristic acid interacted differently with the target proteins. Each protein has a unique interaction profile with different residues depending on the ligand bound to it. This interaction is depicted in Figure 11 and Table 7.

**TABLE 7:** Representation of amino acid residues involved in binding between proteins and three selected metabolites.

Pdb id	Metabolite name	H-bond	Polar bond	Hydrophobic bond
2Z5X	Stearic acid	Ala_68, Tyr_69	Thr_336, Thr_211, Ser_209, Gln_215	Met_445 Tyr_444, Ala_68, Tyr_69, Tyr_407, Met_350, Phe_352, Ile_335, Leu_337, Val_93, Leu_97, Val_210, Ala_111, Phe_208, Phe_108, Cys_323, Ile_325, Ile_180
4COF	Palmitic acid	Arg_207	Thr_202, Ser_156	Val_198, Phe_200, Ala_201, Tyr_205, Tyr_157, Tyr_97, Leu_99
4XUC	Stearic acid	Tyr_121, Val_92	Ser_122, Gln_170, Ser_169, Asn_91, Hie_192	Ala_117, Tyr_118, Cys_119, Tyr_121, Ala_168, Val_92, Met_90, Trp_193, Ile_141, Phe_189
7LIA	Myristic acid	Tyr_175	Asn_177, Thr_439, Ser_438, Thr_497	Phe_335, Tyr_95, Phe_341, Val_343, Ala_169, Ile_172, Ala_173, Trp_103, Tyr_175, Tyr_176, Leu_443, Leu_99

#### 3.11.3 Protein RMSD and ligand RMSD

Root Mean Square Deviation (RMSD) is one of the major parameters used in molecular modeling to determine the structural integrity of protein/ligand complexes. The average RMSD values for the protein/ligand complexes are as follows: 5.398 Å for the 2Z5X-stearic acid complex, 4.863 Å for the 4COF-palmitic acid complex, 1.742 Å for the 4XUC-stearic acid complex, and 3.159 Å for the 7LIA-myristic acid complex. In the 2Z5X-stearic acid complex, the maximum RMSD value was 6.93 Å (frame 719) and the minimum RMSD value was 1.301 Å (frame 1). The maximum (RMSD) values for the other complexes were 6.917 Å for the 4COF-palmitic acid complex (frame 877), 2.335 Å for the 4XUC-stearic acid complex (frame 862), and 4.077 Å for the 7LIA-myristic acid complex (frame 982). The minimum RMSD values were 1.999 Å (frame 3), 0.986 Å (frame 11), and 1.317 Å (frame 1), respectively. It is important to note that the 4XUC-stearic acid complex had the lowest level of RMSD fluctuations, suggesting the highest level of structural stability among the four complexes. The 7LIA-myristic acid complex showed a significant increase in RMSD fluctuations to a maximum of 99 ns, followed by stabilization for the rest of the simulation time. On the other hand, the 4COF-palmitic acid complex showed a high level of instability with significant fluctuations at 7–30 ns and again at 100 ns to the end of the simulation.

**FIGURE 12:**
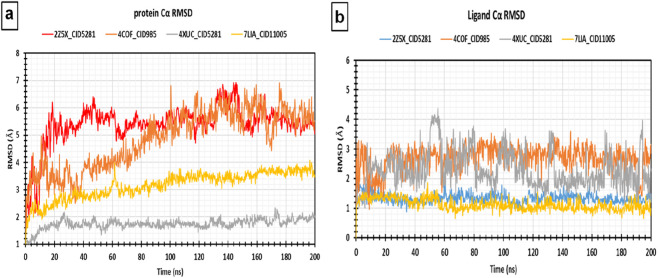
The figure illustrates Protein RMSD **(a)** and Ligand RMSD **(b)**, highlighting the overall structural stability of the complexes throughout the simulation period.

Likewise, the 2Z5X-stearic acid complex had significant oscillations between 6–20 ns and 132–151 ns, followed by stable instability (Figure 12a). Taken together, these findings suggest the presence of varying degrees of structural stability, with 4XUC-stearic acid and 7LIA-myristic acid being more stable than the others.

Ligand RMSD is a measure of ligand stability in the binding pocket, with higher values reflecting higher instability and lower binding affinity. The average ligand RMSD values for the 200 ns simulation were 1.333 Å for 2Z5X-stearic acid, 2.698 Å for 4COF-palmitic acid, 2.309 Å for 4XUC-stearic acid, and 1.123 Å for 7LIA-myristic acid (Figure 12b). In drug development, ligand RMSD values of <2 Å are generally considered to reflect acceptable ligand stability. Based on this criterion, the ligands in the 2Z5X-stearic acid and 7LIA-myristic acid complexes were found to be more stable than those in the 4COF-palmitic acid and 4XUC-stearic acid complexes.

#### 3.11.4 Protein RMSF

**FIGURE 13:**
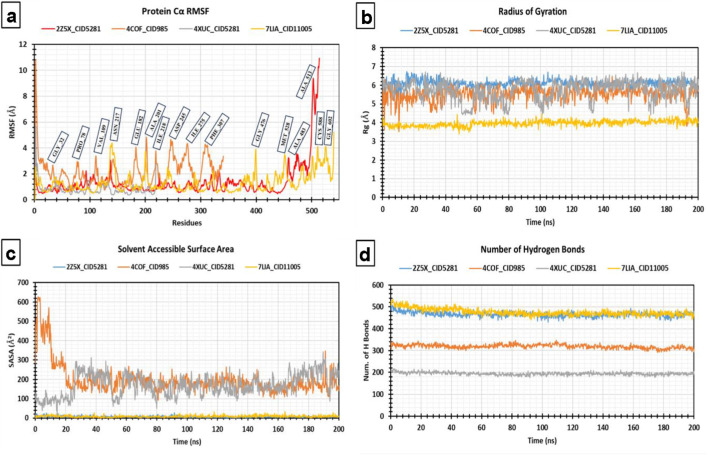
presents a comprehensive analysis of the protein-ligand complexes through four key metrics: protein Root Mean Square Fluctuation (RMSF) in panel **(a)**, Radius of Gyration (Rg) in panel **(b)**, Solvent Accessible Surface Area (SASA) in panel **(c)**, and hydrogen bond counts in panel **(d)**. These visualizations collectively highlight the structural flexibility, conformational compactness, solvent-exposed surface area, and intermolecular binding stability of the complexes during the simulation.

Root Mean Square Fluctuation (RMSF) is a valuable metric for identifying flexible regions in proteins during molecular dynamics simulations, offering insights into residue-level dynamics and the effects of ligand binding. For the protein-ligand complexes, the minimum RMSF values were 0.481 Å (at GLY_25) for 2Z5X-stearic acid, 0.625 Å (at GLN_65) for 4COF-palmitic acid, 0.366 Å (at VAL_188) for 4XUC-stearic acid, and 0.510 Å (at TRP_182) for 7LIA-myristic acid. The maximum RMSF values were 10.930 Å (at LEU_524) for 2Z5X-stearic acid, 10.564 Å (at GLN_1) for 4COF-palmitic acid, 5.605 Å (at ASN_48) for 4XUC-stearic acid, and 5.214 Å (at PRO_617) for 7LIA-myristic acid. The average RMSF values across the 200 ns simulation were 1.352 Å for 2Z5X-stearic acid, 2.175 Å for 4COF-palmitic acid, 0.809 Å for 4XUC-stearic acid, and 1.335 Å for 7LIA-myristic acid (Figure 13a). The most significant fluctuations occurred at protein peak regions involving residues such as GLY_32, PRO_78, VAL_109, ASN_217, GLU_182, ALA_201, ILE_218, ASP_245, ILE_275, PHE_307, GLY_476, MET_528, ALA_483, ALA_513, CYS_588, and GLY_602, highlighting areas of higher flexibility across the complexes.

#### 3.11.5 Radius of Gyration (Rg)

The Radius of Gyration (Rg) is a measure of the distribution of atoms around the central axis of a protein-ligand complex and is an important indicator of structural compactness and conformational dynamics. The Rg values of the four complexes changed over the course of the 200 ns simulation (Figure 13b). In the case of the 2Z5X-stearic acid complex, the Rg values ranged from 5.25 Å (frame 51) to 6.727 Å (frame 77). The 4COF-palmitic acid complex had Rg values ranging from 3.551 Å (frame 52) to 6.468 Å (frame 728). The 4XUC-stearic acid complex had Rg values ranging from 4.401 Å (frame 971) to 6.699 Å (frame 948), while the 7LIA-myristic acid complex had Rg values ranging from 3.388 Å (frame 243) to 4.464 Å (frame 505). The average Rg values were 6.109 Å for 2Z5X-stearic acid, 5.523 Å for 4COF-palmitic acid, 5.701 Å for 4XUC-stearic acid, and 3.961 Å for 7LIA-myristic acid. It is important to note that the 2Z5X-stearic acid and 7LIA-myristic acid complexes had relatively stable Rg values, indicating stable structural compactness. In contrast, the 4COF-palmitic acid and 4XUC-stearic acid complexes exhibited greater fluctuations, indicative of reduced conformational stability.

#### 3.11.6 Solvent Accessible Surface Area (SASA)

Solvent Accessible Surface Area (SASA) is a measure of the protein surface area that is accessible to solvent molecules. The SASA values varied from 0 to 626 Å^2^ in the complexes, depending on the solvent accessibility of certain residues (Figure 13c). The average SASA values for the complexes were 7.050 Å^2^ for 2Z5X-stearic acid, 198.386 Å^2^ for 4COF-palmitic acid, 171.266 Å^2^ for 4XUC-stearic acid, and 8.585 Å^2^ for 7LIA-myristic acid. The significantly lower average SASA values for the 2Z5X-stearic acid and 7LIA-myristic acid complexes indicate that the ligands are more stable in the binding pocket, which could result in stronger protein-ligand binding.

#### 3.11.7 Hydrogen bond analysis

Hydrogen bond analysis plays an important role in understanding molecular interactions and binding stability in drug molecules. In the 200 ns simulation, all complexes showed multiple hydrogen bonds, with a total number ranging from 177 to 535 in the systems (Figure 13d). The average hydrogen bonds were 465.886 for 2Z5X-stearic acid, 319.129 for 4COF-palmitic acid, 194.797 for 4XUC-stearic acid, and 476.562 for 7LIA-myristic acid. The highest average hydrogen bonds in the 2Z5X-stearic acid and 7LIA-myristic acid complexes suggest higher stability and stronger intermolecular interactions with their target proteins.

#### 3.11.8 Protein-ligand (P-L) contacts

Protein-ligand (P-L) interactions play a pivotal role in understanding the binding affinity, stability, and specificity of ligands for their target proteins. To gain a deeper insight into these interactions, Simulation Interaction Diagrams (SID) were created for the four complexes: 2Z5X-stearic acid, 4COF-palmitic acid, 4XUC-stearic acid, and 7LIA-myristic acid, on the 200 ns molecular dynamics simulation trajectory.

**FIGURE 14:**
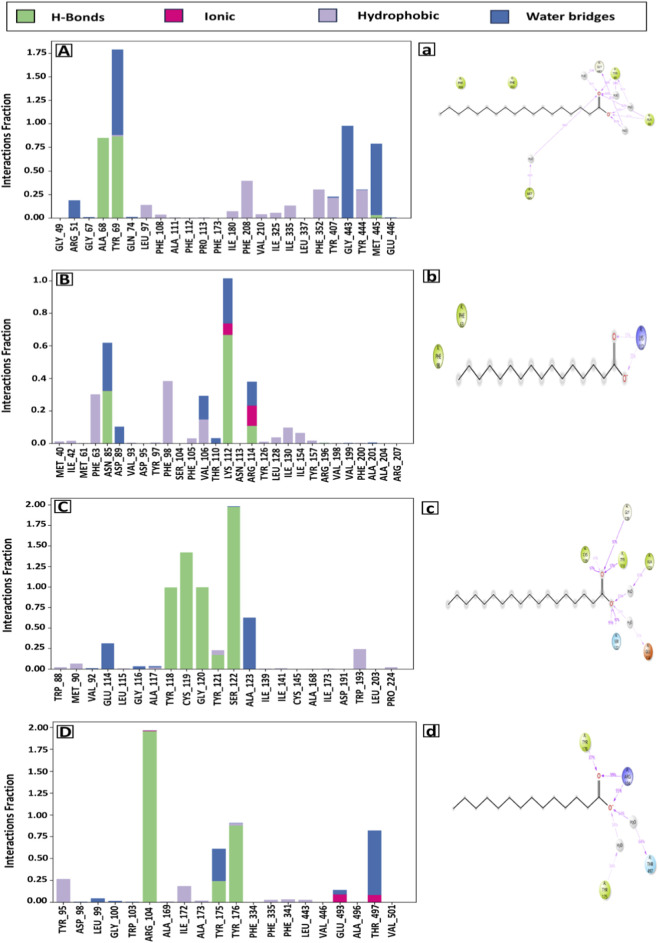
The Figure presents protein-ligand interaction profiles from the 200 ns simulation as stacked bar charts (**A**–**D**) and corresponding 2D interaction diagrams (**a**–**d**) for the complexes: 2Z5X_CID5281, 4COF_CID985, 4XUC_CID5281, and 7LIA_CID11005.

The 2Z5X-stearic acid complex had multiple stable interactions, particularly with TYR_69 (interaction fraction, IF = 1.77) and MET_445 (IF = 0.75) (Figure 14a). In the 4COF-palmitic acid complex, the major interactions included those of ASN_85 (IF = 0.62), LYS_112 (IF = 1.0), and ARG_114 (IF = 0.37) (Figure 14b). The 4XUC-stearic acid complex had fewer stable interactions but maintained a consistent level of contact within the binding pocket throughout the simulation (Figure 14c). In contrast, the 7LIA-myristic acid complex had strong and varied interactions, particularly with ARG_104 (IF = 1.78), TYR_175 (IF = 0.67), and TYR_176 (IF = 0.96) (Figure 14d).

Based on the stability and frequency of these interactions, the 4COF-palmitic acid and 7LIA-myristic acid complexes demonstrated the most robust and persistent protein–ligand binding profiles.

#### 3.11.9 Principal Component Analysis (PCA) and DCCM

**FIGURE 15:**
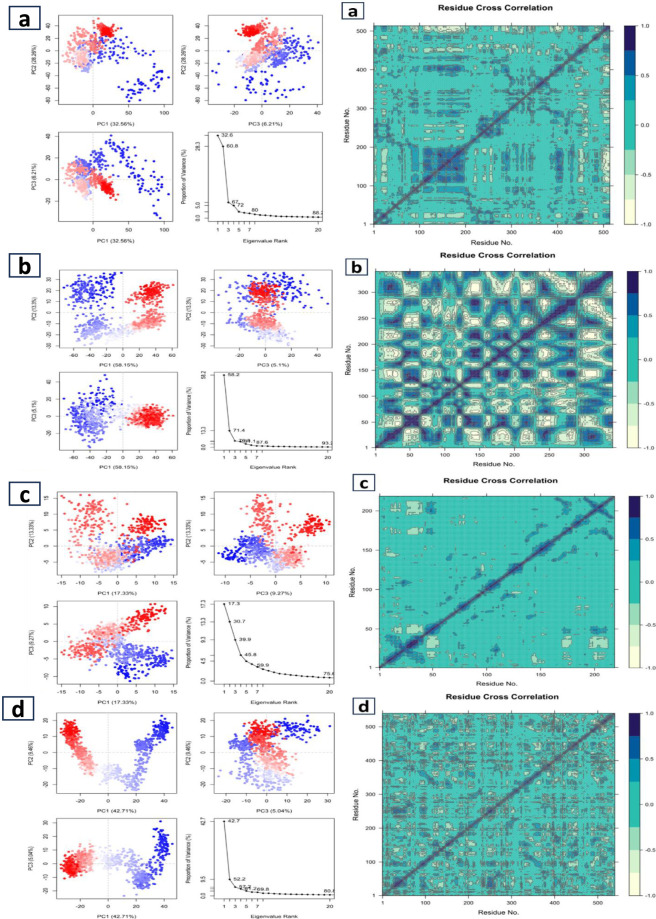
The figure depicts the PCA eigenvalue variation percentages for 2Z5X_CID5281 **(a)**, 4COF_CID985 **(b)**, 4XUC_CID5281 **(c)**, and 7LIA_CID11005 **(d)**, alongside DCCM correlation maps showing interaction dynamics for the same complexes: 2Z5X_CID5281 **(a)**, 4COF_CID985 **(b)**, 4XUC_CID5281 **(c)**, and 7LIA_CID11005 **(d)**.

Principal Component Analysis (PCA) is a technique that provides insightful information on the dynamic behavior and collective internal dynamics of protein-ligand complexes during molecular dynamics simulations. In this study, PCA was used to analyze the atomic fluctuations of the protein-ligand complexes by plotting the eigenvalues against the eigenvector indices (modes) for the top 20 principal components. As shown in Figure 15B, the 4COF-palmitic acid complex had the highest eigenvalues for the top eigenvectors, suggesting that these principal components made a significant contribution to the overall system dynamics. The first five eigenvectors explained 58.2%–87.6% of the total variance, highlighting their dominant role in conformational changes. More specifically, in the case of this complex, PC1 accounted for 58.15%, PC2 for 13.3%, and PC3 for 5.1%, while the relatively lower variance in the higher components indicated a stable and compact conformation. By contrast, the 2Z5X-stearic acid complex (Figure 15A) presented a relatively balanced distribution of dynamics, with PC1 accounting for 32.56%, PC2 for 28.26%, and PC3 for 6.21%. The 4XUC-stearic acid complex (Figure 15C) presented a higher dispersion, with contributions of 17.33% (PC1), 13.33% (PC2), and 9.27% (PC3), which are characteristic of higher flexibility and lower concentrated dynamics. The 7LIA-myristic acid complex (Figure 15D) presented high variability in PC1 (42.71%), with moderate contributions from PC2 (9.46%) and PC3 (5.04%). Color-coded projections in Figures 15A–D show the dynamic profiles of residue mobility, where blue indicates highly flexible residues, white indicates moderately mobile residues, and red indicates rigid residues. The dynamic cross-correlation matrices (DCCM) analysis showed that the 4COF-palmitic acid complex (Figure 15B) had the highest correlated motions, as indicated by the highest cross-correlation coefficients. In the DCCM, blue colors indicate positively correlated motions, while sea green indicates anti-correlated motions. The 4COF-palmitic acid complex had the highest number of positively correlated residue pairs between the ligand (palmitic acid) and the target protein (4COF), indicating highly stable interactions compared to the other complexes.

## 4 Discussion

Anxiety disorders are a worldwide health burden affecting approximately 4.4% of global populations (359 million for 2021) and are expected to increase to 4565.65 per 100,000 by 2030, causing significant morbidity and a decrease in life quality (Bie et al., 2024; Wang et al., 2025; Wu et al., 2025). Current therapeutic approaches involve the use of synthetic metabolites, such as benzodiazepines and Selective Serotonin Reuptake Inhibitors (SSRIs), which immediately increase GABAergic and serotonergic neurotransmission (Rickels and Moeller, 2019; Balon and Starcevic, 2020). However, benzodiazepines promote tolerance, dependence, cognitive deficits, and paradoxical reactions with chronic administration (Fava et al., 2015). SSRIs, although less toxic with long-term use, are often associated with delayed action, persistent adverse effects, and poor sustained response rates (Fornaro et al., 2019; Vahid-Ansari et al., 2019; Domschke et al., 2024).

Oxidative stress is widely recognized as a psychophysiological stressor in anxiety pathophysiology; it is based on the imbalance between ROS and antioxidants, leading to neuroinflammation, neurotransmitter disruption, neurogenesis impairment (Bouayed et al., 2009; Fedoce et al., 2018). Since the brain has high rates of oxygen consumption and lipid levels, it becomes vulnerable to redox damage, leading to the worsening of psychiatric symptoms via serotonergic, GABAergic, or inflammatory pathways (Hovatta et al., 2010; Correia et al., 2023). The above neurological problems make natural antioxidants from plant sources a promising therapy with fewer side effects, including anxiolytic effects, attenuation of ROS, and modulatory effect on the above mechanisms (Gautam et al., 2012; Wang et al., 2023).

The current study establishes the antioxidant and anxiolytic properties of hydroalcoholic extracts and solvent fractions of *B. persicum* fruits, particularly the ethyl acetate fraction and its isolated saturated fatty acids (myristic, palmitic, and stearic acids). By using a bioactivity-guided approach, we assessed the *in-vitro* antioxidant activity, isolated bioactive metabolites, *in-vivo* anxiolytic activity in Swiss albino mice, and used computational modeling to understand molecular interactions with anxiety-related targets. These results are consistent with the increasing trend of using natural antioxidants as safer alternatives for the treatment of anxiety, due to the well-established relationship between oxidative stress and psychiatric disorders (Bouayed et al., 2009; Hovatta et al., 2010; Correia et al., 2023).

Quantitative analysis revealed that the ethyl acetate fraction (EBP) contained the highest amount of TPC (337.408 mg GAE/g) and TFC (286.665 mg RU/g), followed by the butanol fraction (BBP), dichloromethane fraction (DBP), and hexane fraction (HBP). These results are higher than those reported for the essential oil and methanolic extracts of *B. persicum*, which had TPC values of 50–200 mg GAE/g and TFC values of from 20 to 150 mg RU/g (Nickavar et al., 2014; Majidi et al., 2020). The better extraction in the mid-polarity solvent ethyl acetate indicates a preference for the partitioning of polar phenolics and flavonoids, which supports the yield differences in Apiaceae species based on solvent selection (Benmeddour et al., 2013; Idris et al., 2017; Ohikhena et al., 2018).

Column chromatography of EBP resulted in the isolation of three saturated fatty acids: myristic (BP-ET-50), palmitic (BP-ET-100), and stearic (BP-ME-80) acids, which were identified by ^1^H NMR and ^13^C NMR spectroscopy. Spectral data matched literature values (Majó et al., 2021); HMDB0000806 for myristic; HMDB0000220 and PubChem CID_985 for palmitic; HMDB0002784 and PubChem CID_5281 for stearic). While *B. persicum* is known for monoterpenes like γ-terpinene and cuminaldehyde, fatty acids have been underreported; our isolation complements prior detections in seed oils (RC and Kakkalameli, 2025). These long-chain fatty acids may contribute to the observed bioactivities, as saturated fatty acids exhibit antioxidant and anti-inflammatory effects (Hajhashemi et al., 2011).

*In-vivo* anxiolytic studies using the isolated stearic acid (BP-ME-80) from the ethyl acetate fraction of *B*. *persicum* demonstrated robust, dose-dependent activity in both acute and chronic regimens. In the elevated plus maze (EPM), acute oral administration (5 mg/kg) significantly increased open-arm entries (6.16 ± 0.307** vs. 4.16 ± 0.477 in controls) and time spent in open arms (159.5 ± 5.110** sec vs. 98.16 ± 11.167 s), while decreasing closed-arm time, effects comparable to diazepam (0.5 mg/kg). These improvements were sustained and even enhanced upon repeated dosing on days 3 and 7 (open-arm entries up to 7.0 ± 0.577*, time 172.5 ± 5.252** sec), indicating lack of tolerance, a clear advantage over classical benzodiazepines (Fava et al., 2015; Rickels and Moeller, 2019). Similarly, in the light–dark arena (LDA) test, stearic acid markedly increased time spent in the light chamber (171.16 ± 9.365** sec acute; up to 192.16 ± 4.679** sec chronic) and number of transitions, again matching or approaching diazepam performance (185.166 ± 5.186** sec).

Notably, these effects surpass those previously reported for *B. persicum* essential oils in seizure models (Mandegary et al., 2012) and align with fatty acid-mediated neuroprotection in anxiety-like behaviours (Hajhashemi et al., 2011; Gautam et al., 2012; Wang et al., 2023). Similar anxiolytic potential has been documented across the Umbelliferae (Apiaceae) family, yet often requiring higher doses: essential oil of *Foeniculum vulgare* (100–200 mg/kg) and methanolic extracts of *Angelica archangelica* (400 mg/kg) increased open-arm parameters comparably to diazepam (Mesfin et al., 2014; Kumar et al., 2024), while hydroalcoholic extracts of *Pimpinella anisum* (100–200 mg/kg) and polyphenolic fractions of *Carum carvi* (50–100 mg/kg) enhanced light-chamber time and exploratory activity (Es-safi et al., 2021; Shahamat et al., 2016). The oleo-gum resin of *Ferula assa-foetida* also showed dose-dependent EPM activity (Alqasoumi et al., 2012). These behavioural outcomes occurred without acute toxicity at 2000 mg/kg (OECD 425), reinforcing the safety profile consistent with traditional Persian ethnopharmacological use of *B. persicum* seeds as a calming remedy (Sofi et al., 2009; Zaman et al., 2013).

Computational studies reinforced these observations. Molecular docking showed favorable binding affinities: stearic acid to (MAO) (2Z5X; −7.6 kcal/mol) and COMT (4XUC; −5.4 kcal/mol), palmitic acid to GABA(A)R (4COF; −5.3 kcal/mol), and myristic acid to serotonin transporter (7LIA; −5.7 kcal/mol). These scores are comparable to or better than those for natural anxiolytics against similar targets (Kenda et al., 2022). Key interactions involved hydrophobic contacts and hydrogen bonds with residues like TYR_69 (stearic acid) and ARG_104 (myristic acid). MD simulations (200 ns) confirmed stability, with low RMSD (<4 Å average for most complexes), RMSF (<2.2 Å average), and Rg (4–6 Å), indicating compact conformations. Hydrogen bonds (average 195–477) and P-L contacts (e.g., IF up to 1.78) were persistent, especially in stearic and myristic acids. PCA and DCCM revealed dominant motions in PC1–PC3 (up to 87.6% variance) and strong correlations in 4COF-palmitic, aligning with simulations of fatty acids in neuroprotective contexts (Fedoce et al., 2018; Nunes et al., 2025).

Overall, our results validate *B. persicum’s* antioxidant prowess (Nickavar et al., 2014) and extend its anxiolytic potential, likely via fatty acid modulation of oxidative stress and neurotransmitter targets (Hajhashemi et al., 2011; Hassamal, 2023). Compared to synthetic drugs, these metabolites offer reduced side effects (Kenda et al., 2022), warranting further clinical validation for anxiety management amid rising global prevalence (Javaid et al., 2023; Wu et al., 2025). Limitations include focus on one metabolite for *in-vivo* tests and lack of mechanistic biomarkers; future studies should explore synergies and human trials.

The anxiolytic activity of myristic, palmitic, and stearic acids isolated from the bioactive ethyl acetate fraction of *B. persicum* stems from their potent antioxidant capacity and multi-target modulation of anxiety-related proteins. These fatty acids effectively scavenge ROS, thereby attenuating oxidative stress and neuroinflammation, while molecular docking and 200 ns MD simulations confirmed stable binding (ΔG° = −5.3 to −7.6 kcal/mol; RMSD < 2.7 Å) to MAO-A, GABA(A) receptor β3, COMT, and SERT through hydrogen bonds and hydrophobic interactions. This synergistic action restores GABAergic/serotonergic balance, directly explaining the significant behavioral improvements in elevated plus maze and light–dark arena tests (P < 0.01–0.001 vs. control), comparable to diazepam. These findings mechanistically validate the longstanding traditional Persian use of *B. persicum* seeds as a calming and anxiolytic remedy for stress-related disorders.

## 5 Conclusion

This study demonstrates the antioxidant and anxiolytic potential of *B. persicum* fruits, particularly the ethyl acetate fraction enriched with saturated fatty acids (myristic, palmitic, and stearic acid). The fraction exhibited notable *in vitro* antioxidant activity, as evidenced by high phenolic (337.408 mg GAE/g) and flavonoid (286.665 mg RE/g) contents, potent DPPH radical scavenging and reducing power capacities. Bioactivity-guided isolation, followed by spectroscopic characterization, confirmed the presence of the three fatty acids. In acute rodent models, stearic acid produced significant anxiolytic-like effects in the elevated plus maze and light–dark arena tests (P < 0.01–0.001 vs. control). Molecular docking and 200 ns MD simulations further supported stable interactions with key anxiety-related targets (MAO-A, GABA(A) receptor β3, COMT, and SERT). These preliminary findings provide mechanistic support for the traditional Persian use of *B. persicum* as a calming medicinal spice.

### 5.1 Limitations

This study provides promising preclinical evidence but has several important limitations. Anxiolytic activity was assessed *in-vivo* only for stearic acid (BP-ME-80) using acute single-dose administration in two behavioral models (elevated plus maze and light–dark arena); myristic and palmitic acids were evaluated solely through *in silico* docking/MD simulations and as components of the active ethyl acetate fraction. No chronic dosing, tolerance, or withdrawal studies were performed, and mechanistic validation relied entirely on computational approaches without complementary *in-vivo* biochemical assays (e.g., GABA binding, serotonin turnover, or ROS levels in brain tissue). Pharmacokinetic and bioavailability data for the isolated fatty acids are absent, and only acute oral toxicity of the crude extract (not the pure compounds) was tested. The work used a single mouse strain and standard acute paradigms; broader models (e.g., chronic stress-induced anxiety) and larger sample sizes would strengthen generalizability. Finally, synergistic interactions among the three fatty acids or with the essential oil fraction were not investigated experimentally.

### 5.2 Future perspectives

Future studies should extend *in vivo* behavioral testing to myristic and palmitic acids (individually and in combination) to clarify potential synergistic or differential effects. Chronic administration protocols, full dose response curves, and interaction studies with standard anxiolytics are needed. Detailed mechanistic work including *ex-vivo* neurotransmitter assays, receptor antagonist reversal experiments, and advanced omics (transcriptomics/proteomics) will elucidate molecular pathways. Pharmacokinetic profiling, nano-formulation development to improve bioavailability, long-term toxicity/safety assessments, and ultimately well-designed human clinical trials are essential for translational success. Exploring combinations with *B. persicum* essential oils or other Apiaceae species could further enhance therapeutic efficacy.

## Data Availability

The original contributions presented in the study are included in the article/Supplementary Material, further inquiries can be directed to the corresponding author.
