# Supplementary material for: Evaluation of free radical scavenging and anxiolytic activities of Bunium persicum (Boiss.) B. Fedtsch. bioactive metabolites through multi-method experimental and computational approaches
Source: Front Pharmacol. 2026 Jun 5;17:1805095. doi: 10.3389/fphar.2026.1805095 (PMC13279422; doi:10.3389/fphar.2026.1805095)

**Evaluation of free radical scavenging and anxiolytic activities of *Bunium persicum* (Boiss.) B. Fedtsch. bioactive metabolites through multi-method experimental and computational approaches**

Riehana Gani^1,2^, Umar Yousuf^3^, Noimul Hasan Siddiquee^4^, Mushtaq A. Mir^5^, Aadil Yaseen^6^, Nasreena Bashir^5^, Venkatramanan Varadharajan^7^, Md. Ifteker Hossain^4^, Pervaize Ahmad Dar^8^, Mohd Younis Dar^1^, Zulfiqar Ali Bhat^2*^, Basharat Ahmad Bhat^9*^

*^1^Regional Research Institute of Unani Medicine (RRIUM), Naseem Bagh Campus, University of Kashmir, Srinagar, J & K, India.*

*^2^Department of Pharmaceutical Sciences, University of Kashmir, Srinagar, J & K, India*

*^3^Centre of Research for Development (CORD), University of Kashmir, Srinagar, J&K, India*

*^4^Department of Microbiology, Noakhali Science and Technology University, Noakhali-3814, Bangladesh*

*^5^Department of Clinical Laboratory, College of Applied Medical Sciences, King Khalid University, Abha, KSA*

*^6^Department of Chemistry, University of Kashmir, Srinagar, J&K, India*

*^7^Department of Zoology, Model Degree College, Charar-i-Sharief, J & K, India*

*^8^Department of Biotechnology, PSG College of Technology, Coimbatore, India*

*^9^Department of Bio-Resources, School of Biological Sciences, University of Kashmir, J*&*K, India*

**Abstract**

Anxiety disorders, linked to oxidative stress and neuroinflammation, represent a major global health burden and have prompted interest in safer plant-derived antioxidants. *Bunium persicum,* a traditional Persian medicinal spice, is valued for its calming and anxiolytic effects. This study evaluated the antioxidant and anxiolytic potential of hydroalcoholic extracts and solvent fractions of *B. persicum* fruits through bioactivity-guided isolation of key metabolites, supported by *in-vivo* and *in-silico* validation of traditional uses. Antioxidant activity was assessed using total phenolic content, total flavonoid content, DPPH radical scavenging, and reducing power assays. The ethyl acetate fraction exhibited the highest phenolic (337.408 mg GAE/g) and flavonoid (286.665 mg RU/g) contents, strongest DPPH scavenging and reducing power capacities. Bioactivity-guided fractionation led to the isolation and identification of three fatty acids: myristic, palmitic, and stearic acid. Stearic acid showed significant anxiolytic effects in Swiss albino mice (elevated plus maze and light–dark arena tests, P < 0.01–0.001 vs. control), comparable to diazepam, with no acute oral toxicity at 2000 mg/kg. Molecular docking and 200 ns MD simulations confirmed stable, favourable binding (ΔGº = –5.3 to –7.6 kcal/mol) to key anxiety targets (Human Monoamine Oxidase (MAO), Human gamma-aminobutyric acid receptor, GABA(A) receptor β3, Catechol-O-methyltransferase (COMT) and Serotonin transporter (SERT) via hydrogen bonding and hydrophobic interactions. These findings mechanistically validate the traditional Persian use of *B. persicum* as a natural anxiolytic agent and highlight its potential for managing anxiety disorders.

**Keywords:** *Bunium persicum*, anxiety disorders, antioxidant activity, anxiolytic effect, fatty acids, molecular docking, molecular dynamics simulation

**Table S1**. DPPH radical scavenging activity of *B. persicum* fruit fractions and ascorbic acid standard

| **Sample** | **Concentration (µg/ml)** | **Absorbance 517nm** | **% scavenging activity** |
| --- | --- | --- | --- |
| Ascorbic acid (AA) | 20 | 0.380 | 29.889 |
|  | 40 | 0.290 | 46.494 |
|  | 60 | 0.203 | 62.546 |
|  | 80 | 0.132 | 75.645 |
|  | 100 | 0.072 | 86.346 |
| Hexane fraction (HBP) | 20 | 0.309 | 28.965 |
|  | 40 | 0.204 | 43.448 |
|  | 60 | 0.168 | 61.379 |
|  | 80 | 0.117 | 73.103 |
|  | 100 | 0.067 | 84.597 |
| Dichloromethane fraction (DBP) | 20 | 0.253 | 17.049 |
|  | 40 | 0.198 | 35.081 |
|  | 60 | 0.101 | 66.885 |
|  | 80 | 0.071 | 76.721 |
|  | 100 | 0.059 | 80.655 |
| Ethyl acetate fraction (EBP) | 20 | 0.190 | 46.778 |
|  | 40 | 0.130 | 63.585 |
|  | 60 | 0.107 | 70.028 |
|  | 80 | 0.057 | 84.033 |
|  | 100 | 0.022 | 93.837 |
| Butanol fraction (BBP) | 20 | 0.199 | 42.074 |
|  | 40 | 0.128 | 63.112 |
|  | 60 | 0.086 | 75.216 |
|  | 80 | 0.052 | 85.014 |
|  | 100 | 0.030 | 90.778 |

**Table S2**. Reducing power ability of various fractions of fruits of *Bunium persicum*

| **Sample** | **Volume (µl)** | **Concentration (µg/ml)** | **A 700nm** |
| --- | --- | --- | --- |
| Butylated Hydroxy Toulene (BHT) | 100 | 20 | 0.576 |
|  | 200 | 40 | 0.989 |
|  | 400 | 80 | 1.843 |
|  | 800 | 160 | 2.669 |
|  | 1600 | 320 | 4.268 |
| Hexane fraction (1 mg/ml) of *B. persicum* | 100 | 20 | 0.073 |
|  | 200 | 40 | 0.125 |
|  | 400 | 80 | 0.245 |
|  | 800 | 160 | 0.420 |
|  | 1600 | 320 | 0.693 |
| DCM fraction (1 mg/ml) of *B. persicum* | 100 | 20 | 0.135 |
|  | 200 | 40 | 0.248 |
|  | 400 | 80 | 0.417 |
|  | 800 | 160 | 0.805 |
|  | 1600 | 320 | 1.234 |
| Ethyacetate fraction (1 mg/ml) of *B. persicum* | 100 | 20 | 0.332 |
|  | 200 | 40 | 0.611 |
|  | 400 | 80 | 1.090 |
|  | 800 | 160 | 1.459 |
|  | 1600 | 320 | 2.507 |
| Butanol fraction (1 mg/ml) of *B. persicum* | 100 | 20 | 0.251 |
|  | 200 | 40 | 0.407 |
|  | 400 | 80 | 0.707 |
|  | 800 | 160 | 1.204 |
|  | 1600 | 320 | 1.689 |

**Table S3**. Anxiety related behavior using elevated plus maze model

| **Treatment** | **Days** | **Time spent in seconds** | | | **Number of entries** | |
| --- | --- | --- | --- | --- | --- | --- |
|  |  | **Latency** | **Open arm** | **Closed arm** | **Open arm** | **Closed arm** |
| **Control** | Day 1 | 64.33 ± 3.518 | 98.16 ± 11.167 | 139.16 ±8.348 | 4.16 ± 0.477 | 6.33 ± 0.421 |
|  | Day 3 | 57.66 ± 4.616 | 86.83 ± 3.987 | 155.5 ± 3.658 | 3.16 ± 0.307 | 5.0 ± 0.365 |
|  | Day 7 | 55.5 ± 1.910 | 80.66 ± 4.333 | 163.83 ± 4.968 | 5.0 ± 0.577 | 7.5 ± 0.619 |
| **Diazepam (0.5mg/kg)** | Day 1 | 47 ± 1.966^**^ | 166.5 ± 5.506^**^ | 86.5 ± 5.078^**^ | 5.16 ± 0.307^*^ | 4 ± 0.447^**^ |
|  | Day 3 | 45.5 ± 2.513^*^ | 177.83 ± 7.035^**^ | 76.66 ± 7.961^**^ | 6.83 ± 0.600^**^ | 5 ± 0.365^ns^ |
|  | Day 7 | 45 ± 2.049^*^ | 187.83 ± 6.745^**^ | 67.16 ± 6.210^**^ | 7.16 ± 0.401^*^ | 4.66 ± 0.49^*^ |
| **Test group**  **(BP-ME-80)**  **(5mg/kg)** | Day 1 | 43.33 ± 4.072^**^ | 159.5 ± 5.110^**^ | 92.66 ± 9.885^**^ | 6.16 ± 0.307^**^ | 4.66 ±0.333^*^ |
|  | Day 3 | 46 ± 2.887^ns^ | 165.83 ± 4.615^*^ | 88.16 ± 5.400^**^ | 6 ± 0.516^**^ | 4.83 ± 0.40^ns^ |
|  | Day 7 | 43.33 ± 2.801^**^ | 172.5 ± 5.252^**^ | 84.16 ± 7.026^**^ | 7 ± 0.577^*^ | 4.16 ± 0.30^**^ |

Results are expressed as Mean ± SEM. (n=6)

^*^P < 0.05, ^**^P < 0.01, ^***^P < 0.001, P > 0.05 will be considered as significant, highly significant, extremely significant and insignificant respectively compared with control. Data was statistically analysed by ANOVA followed by Dunnet’s Test.

**Table S4**. Anxiety related behavior using light / dark arena model

| **Treatments** | **Days** | **Time spent in seconds** | | **Number of entries** | |
| --- | --- | --- | --- | --- | --- |
|  |  | **Light** | **Dark** | **Light** | **Dark** |
| **Control** | Day 1 | 113.5 ± 6.228 | 186.5 ± 6.228 | 4.66 ± 0.333 | 5.66 ±0.421 |
|  | Day 3 | 115.33 ± 5.80 | 184.66 ± 5.80 | 5 ± 0.577 | 6.66 ± 0.333 |
|  | Day 7 | 115.16 ± 7.670 | 184. 83 ± 7.670 | 5.16 ± 0.401 | 7 ± 0.447 |
| **Diazepam**  **(0.5mg/kg)** | Day 1 | 185.166±5.186^**^ | 114.83 ± 5.186^**^ | 6.66 ± 0.494^*^ | 5.83 ± 0.477^ns^ |
|  | Day 3 | 190.16 ± 7.46^**^ | 109.83 ± 7.463^**^ | 6.5 ± 0.428^ns^ | 4.83 ± 0.477^*^ |
|  | Day 7 | 190 ± 6.563^**^ | 110 ± 65.63^**^ | 7.16 ± 0.307^*^ | 4.33 ±0.421^**^ |
| **Test group**  **(BP-ME-80)**  **(5 mg/kg)** | Day 1 | 171.16 ± 9.365^**^ | 128.83 ± 9.365^**^ | 6.83 ± 0.477^**^ | 5.66 ± 0.557^ns^ |
|  | Day 3 | 184.5 ± 5.175^**^ | 115.5 ± 5.175^**^ | 7.66 ± 0.421^**^ | 6.16 ± 0.473^ns^ |
|  | Day 7 | 192.16 ± 4.679^**^ | 107.83 ± 4.67^**^ | 7.83 ± 0.792^**^ | 5.83 ± 0.307^ns^ |

Results are expressed as Mean ± SEM. (n=6)

^*^P<0.05, ^**^P<0.01, ^***^P<0.001, P>0.05 will be considered as significant, highly significant, extremely significant and insignificant respectively compared with control. Data was statistically analysed by ANOVA followed by Dunnet’s Test.

**Supplementary figure 1(a-c)**

**^1^H NMR** (500 MHz, Chloroform) δ 9.52 (s, 1H), 2.30 – 2.26 (m, 2H), 1.65 – 1.61 (m, 2H), 1.39 – 1.21 (m, 20H), 1.01 – 0.97 (m, 3H).


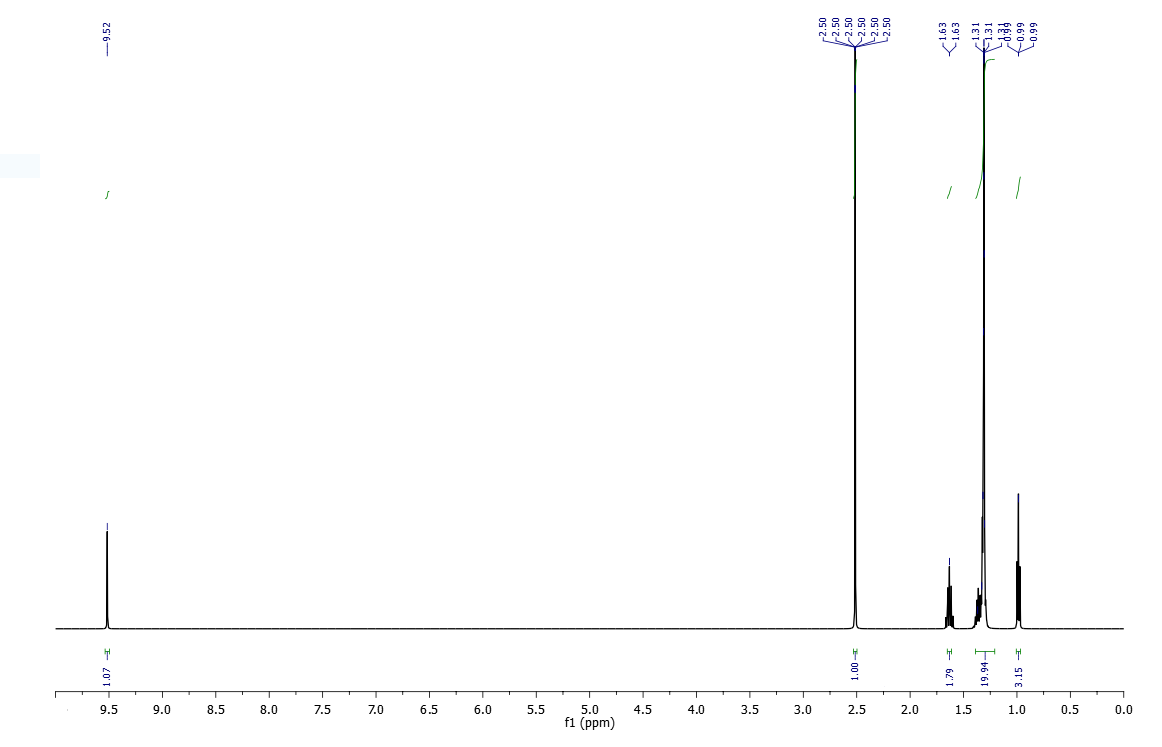


**^13^C NMR** (125 MHz, Common NMR Solvents) δ 177.13 (s), 34.35 (s), 31.64 (s), 29.01 (d, *J* = 13.4 Hz), 24.80 (s), 22.93 (s), 14.01 (s).

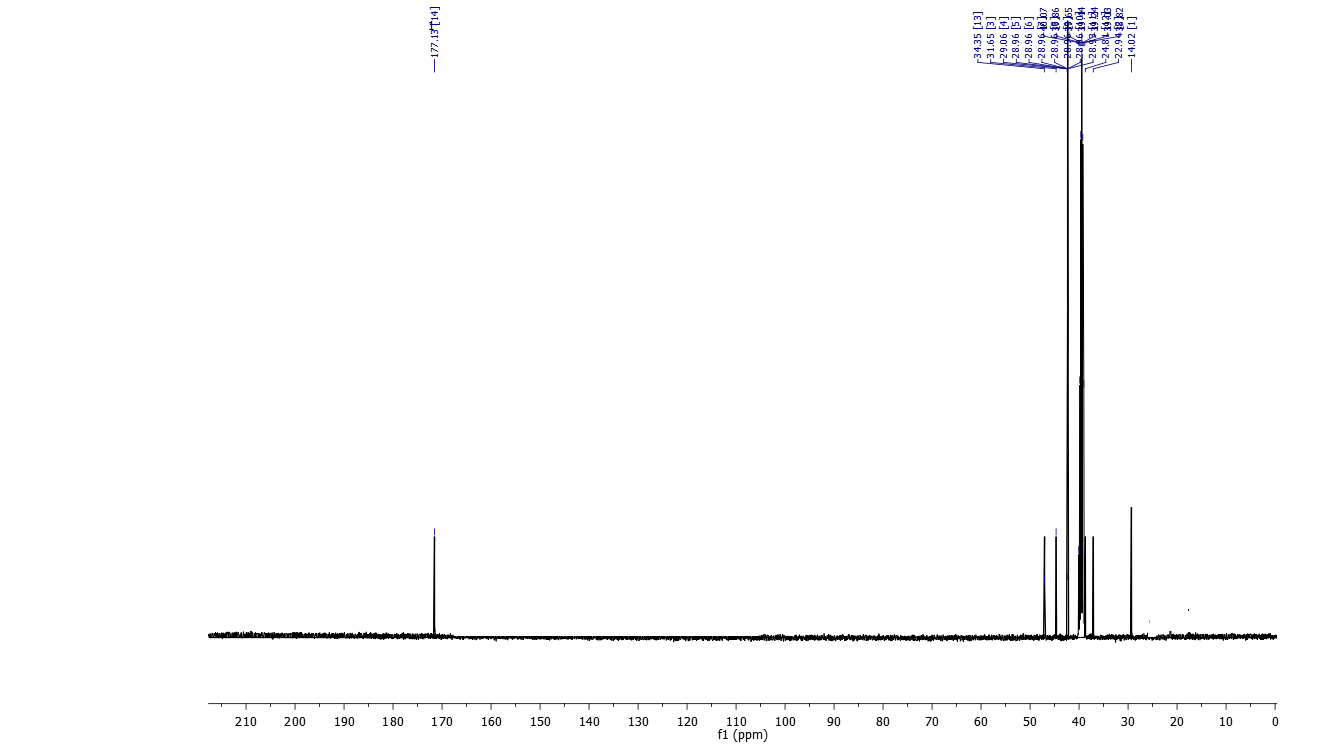


**^1^H NMR** (500 MHz, Chloroform) δ 9.52 (s, 3H), 2.30 – 2.26 (m, 6H), 1.65 – 1.61 (m, 5H), 1.39 – 1.21 (m, 7H), 1.01 – 0.97 (m, 9H).

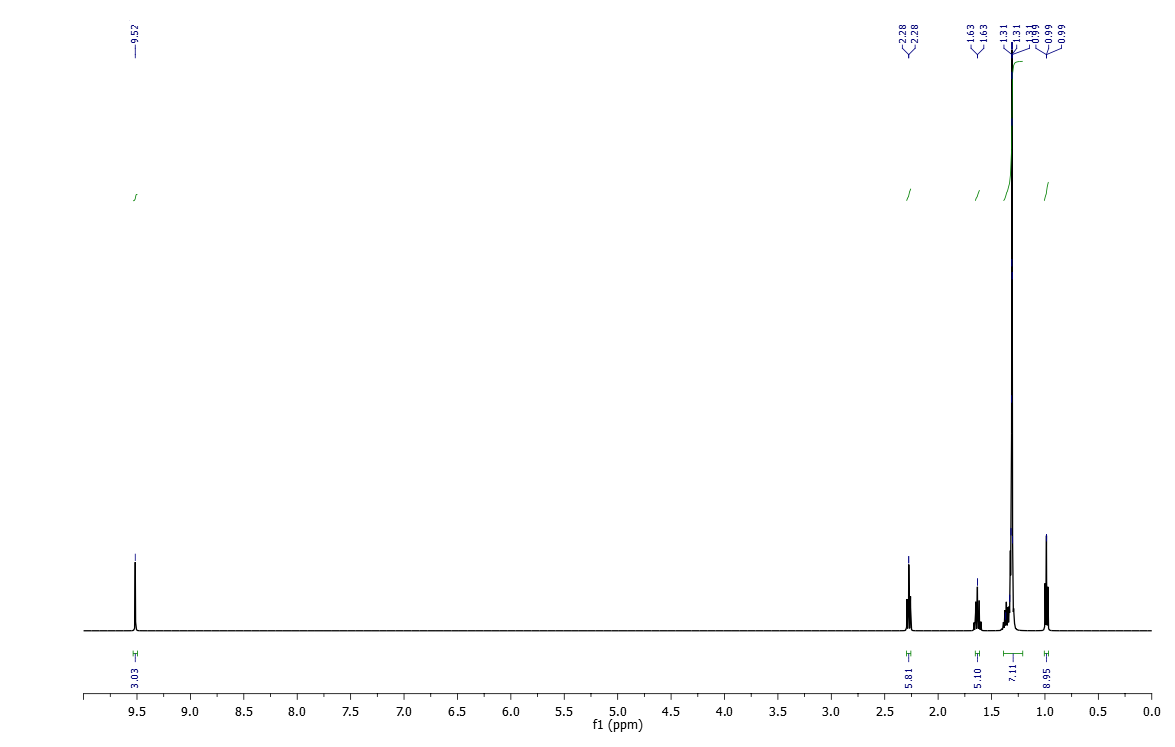


**^13^C NMR** (125 MHz, Common NMR Solvents) δ 177.13 (s), 34.35 (s), 31.65 (s), 29.26 – 28.74 (m), 24.81 (s), 22.94 (s), 14.02 (s).

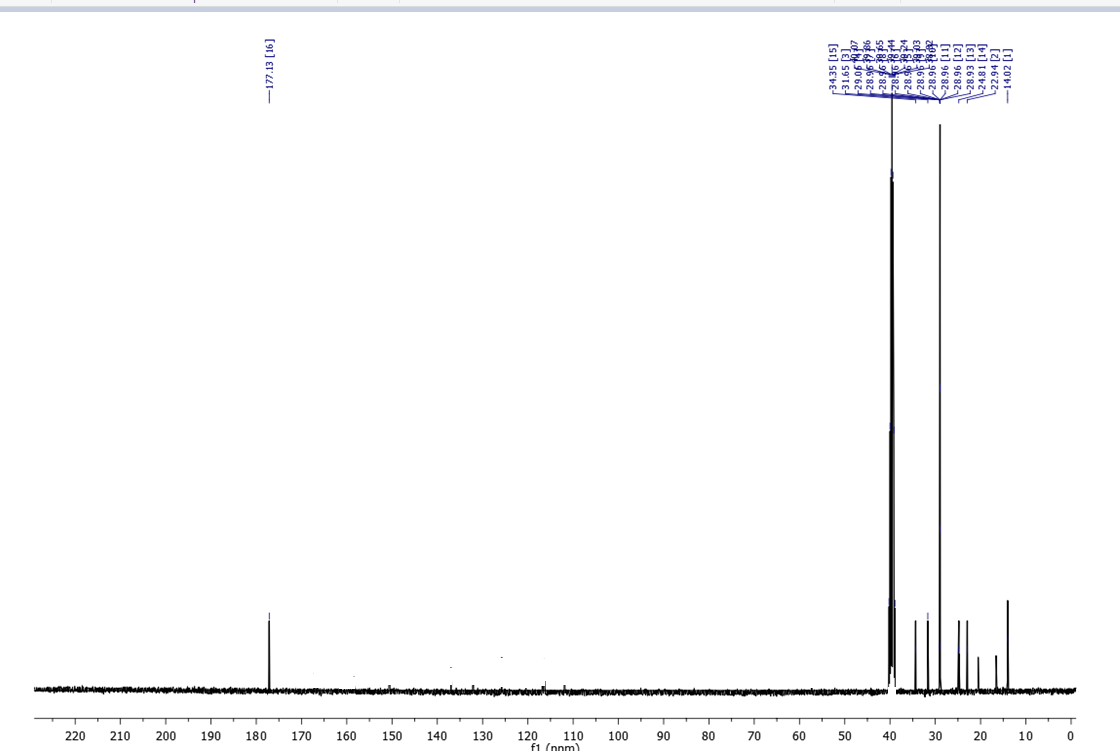


**^1^H NMR** (500 MHz, Chloroform) δ 9.52 (s, 3H), 2.30 – 2.26 (m, 6H), 1.65 – 1.61 (m, 5H), 1.39 – 1.21 (m, 7H), 1.01 – 0.97 (m, 9H).

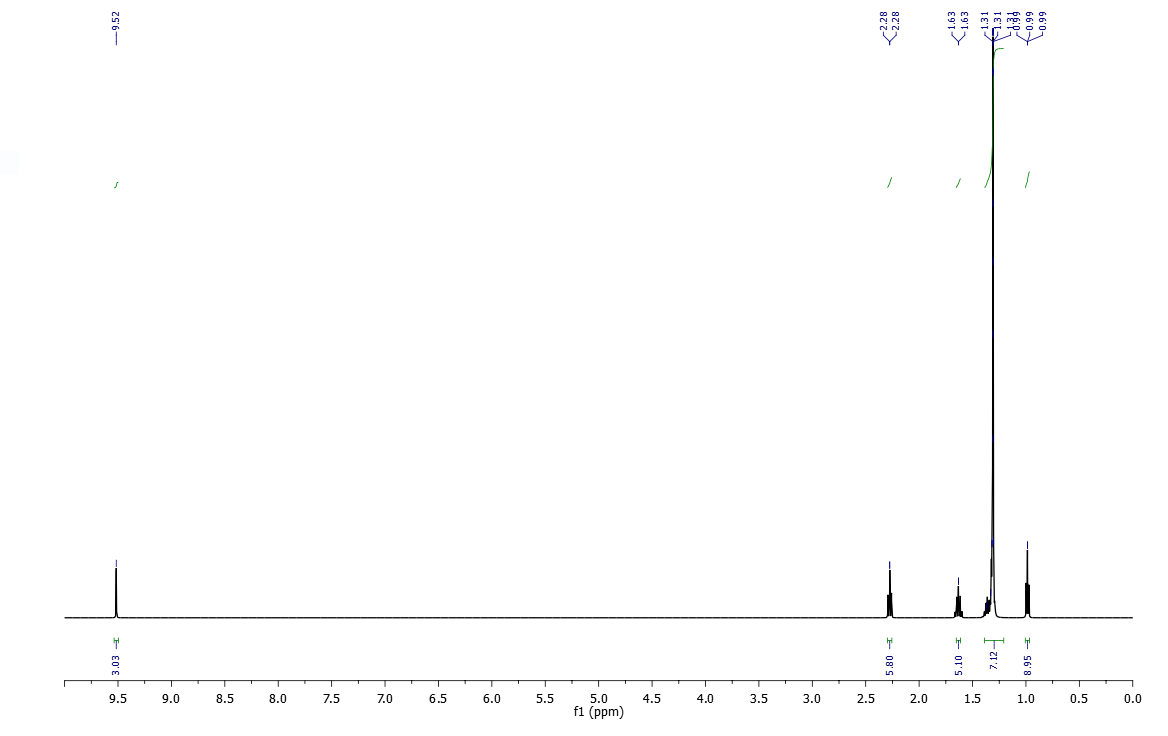


**^13^C NMR** (125 MHz, Common NMR Solvents) δ 177.13 (s), 34.35 (s), 31.65 (s), 29.26 – 28.82 (m), 24.81 (s), 24.09 – 23.69 (m), 22.94 (s), 14.29 – 13.81 (m).

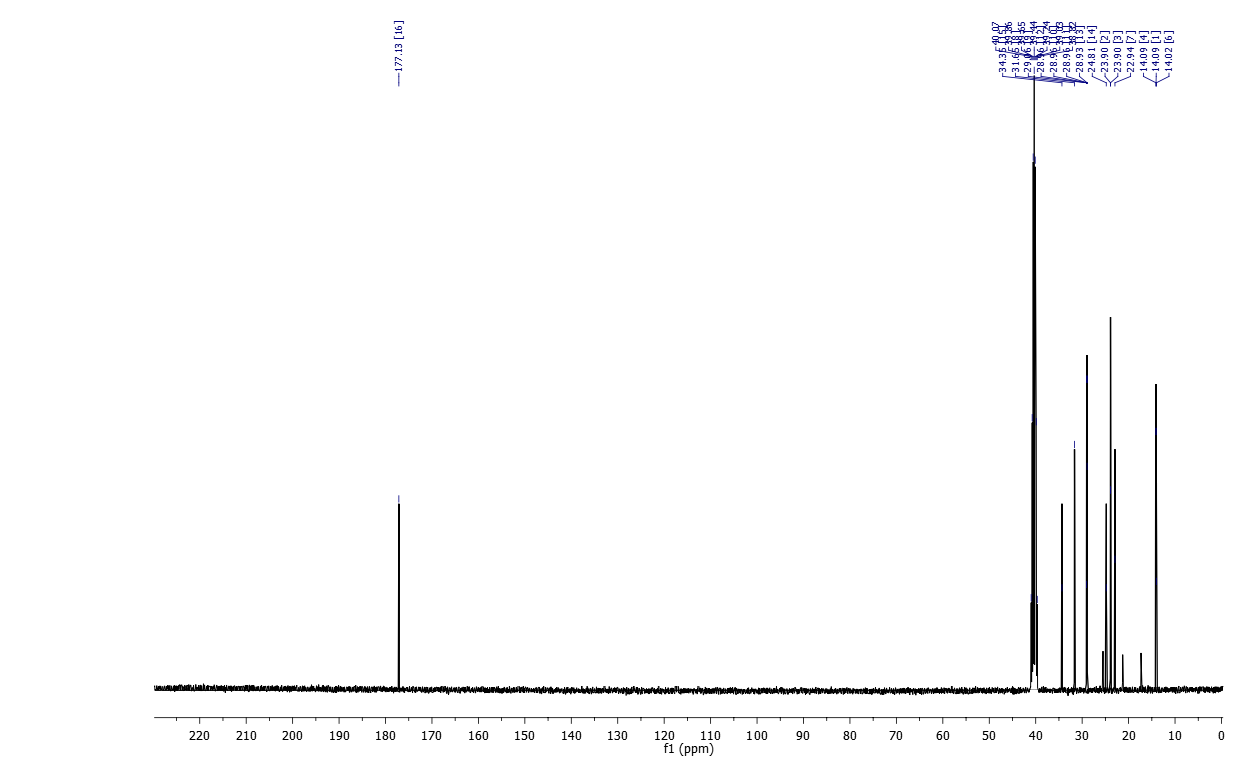

Supplement: Supplementary file 1 [file Supplementaryfile1.docx]
